# Supplementary material for: Aerosol Delivery of Synthetic mRNA to Vaginal Mucosa Leads to Durable Expression of Broadly Neutralizing Antibodies against HIV
Source: Mol Ther. 2020 Jan 10;28(3):805–19. doi: 10.1016/j.ymthe.2020.01.002 (PMC7054722; doi:10.1016/j.ymthe.2020.01.002)
Supplement: Document S1. Figures S1–S6 and Tables S1–S3 [file mmc1.pdf]

YMTHE, Volume *28*

Supplemental Information

Aerosol Delivery of Synthetic mRNA to Vaginal Mucosa Leads to Durable Expression of Broadly Neutralizing Antibodies against HIV

Kevin E. Lindsay, Daryll Vanover, Merrilee Thoresen, Heath King, Peng Xiao, Peres Badial, Mariluz Araínga, Seong Bin Park, Pooja M. Tiwari, Hannah E. Peck, Emmeline L. Blanchard, Jean M. Feugang, Alicia K. Olivier, Chiara Zurla, Francois Villinger, Amelia R. Woolums, and Philip J. Santangelo

Supplementary Materials:

A B C

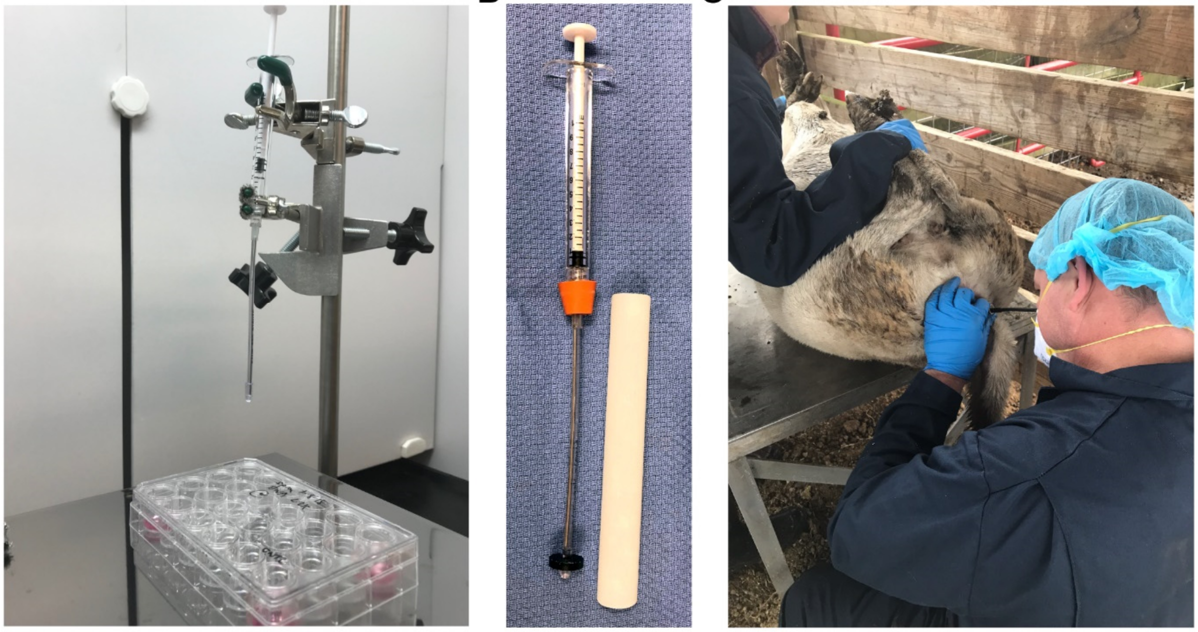

Fig. S1. ***In vitro*** **and** ***in vivo*** **aerosolization apparatus and field application example. (A)** *In vitro* aerosol apparatus. Height was adjusted using a scissor-lift lab jack. Teleflex was held using clamps and actuated by hand. **(B)** *In vivo* Teleflex apparatus with speculum. Teleflex syringe was modified using flat and conical rubber washer to allow for consistent centering and distance when placed in the speculum. The speculum was a cut and sanded piece of plastic tubing. **(C)** Treatment of sheep in field. Sheep were sedated and placed in dorsal recumbency to allow for easy mRNA application.

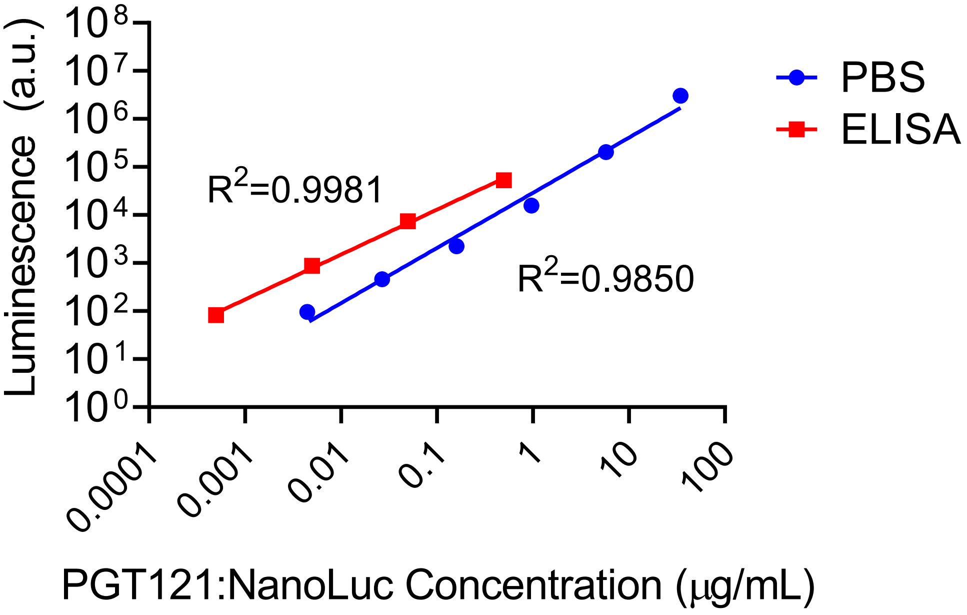

Fig. S2. HIV gp120 ELISA using PGT121:NanoLuc Luminescence. Luminescence of a range of concentrations of purified PGT121:NanoLuc were either measured in solution (PBS) or after capture via gp120 coated plate (ELISA). Linear regression was performed on log-log transformed data.

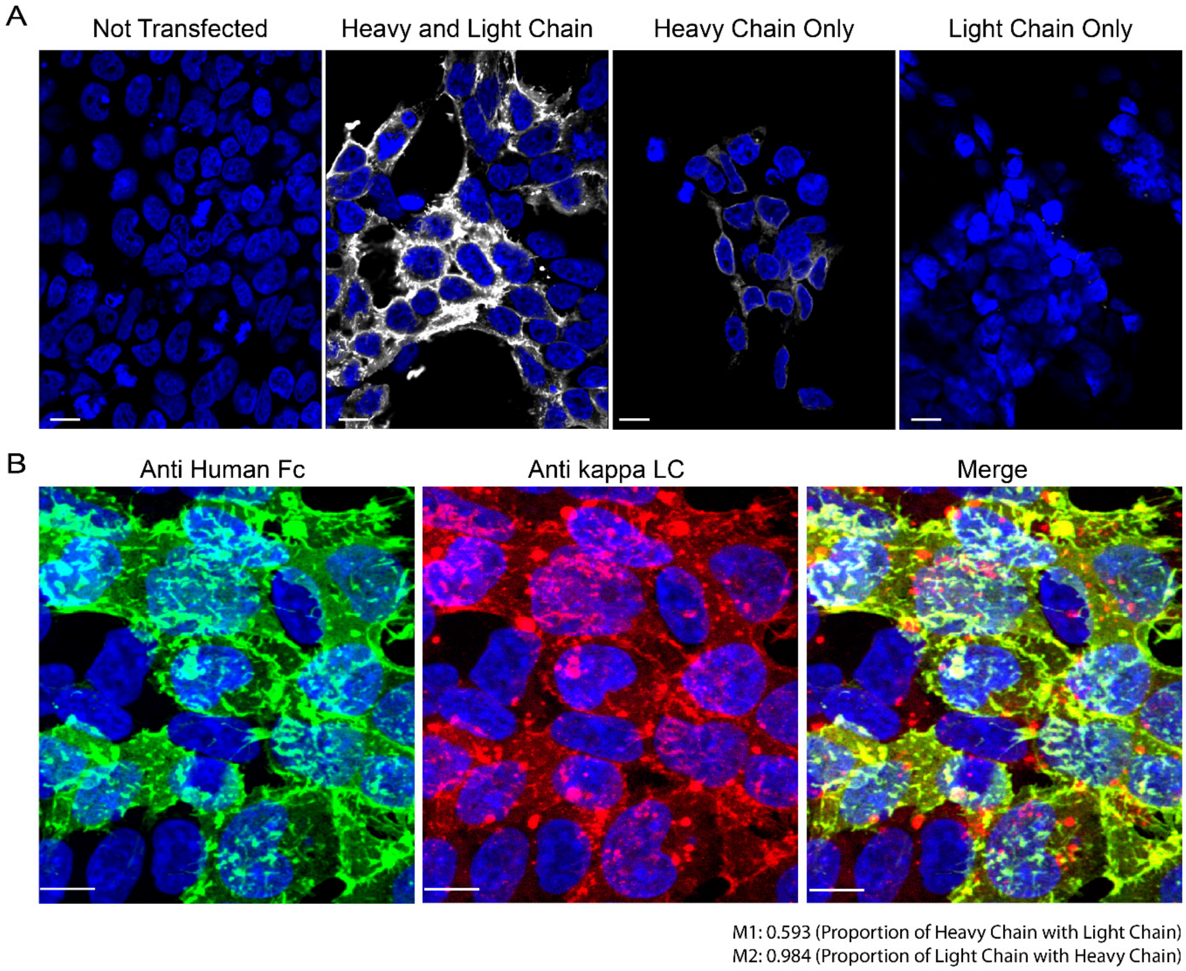

Fig. S3. PGT121 LC and HC localization to cell surface after mRNA transfection. (A) HEK293 cells were transfected using 1ug of total synthetic PGT121 mRNA, in the following conditions: 1) as a 4:1 ratio of HC to LC encoding transcripts; 2) HC encoding transcripts only; or 3) LC encoding transcripts only. 24 hours post-transfection, the cells were fixed, not permeabilized, and immunostained with anti-human antibody (Jackson). White - anti-human antibody (Jackson), Blue -DAPI. Scale bars are 10µm. **(B)** HEK293 cells were transfected with 1ug of HC and LC PGT121 mRNA, at a 4:1 ratio. After 24 hours, the cells were fixed and immunostained with anti-human Fc and anti-kappa light chain antibodies. Colocalization coefficients indicate that 98% of the LC protein overlaps with HC protein. Green – anti-human antibody (Jackson), Red – anti-kappa light chain (BD), Blue – DAPI. Scale bars are 10µm.

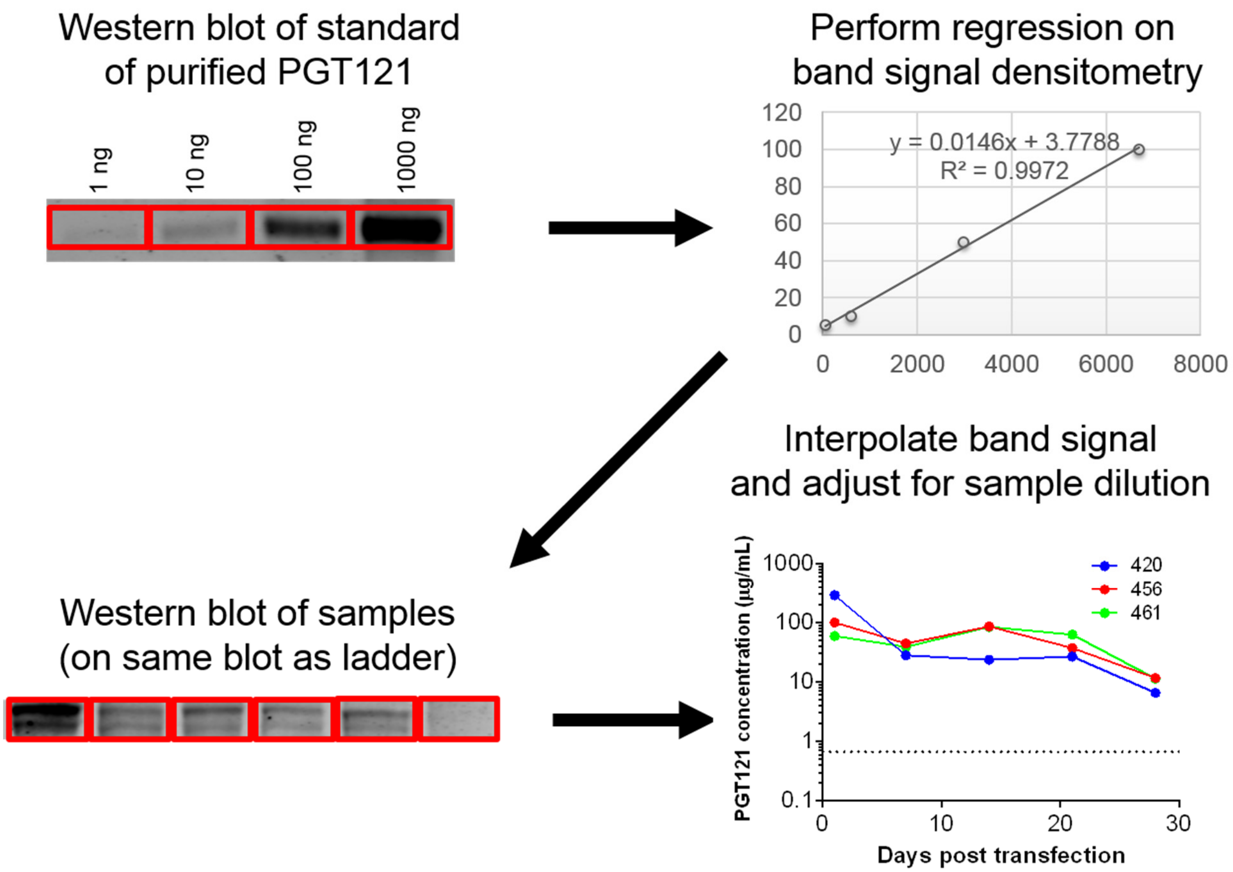

Fig. S4. Quantitative Western methodology for aPGT121-NanoLuc. Schematic of quantitative western blot protocol. On each gel, a standard of purified PGT121 was loaded and quantified by densitometry. A linear regression was then run on these values, and the densitometry of the samples was interpolated to determine the amount of PGT121 in the loaded sample. Samples were loaded onto the same gel in all cases.

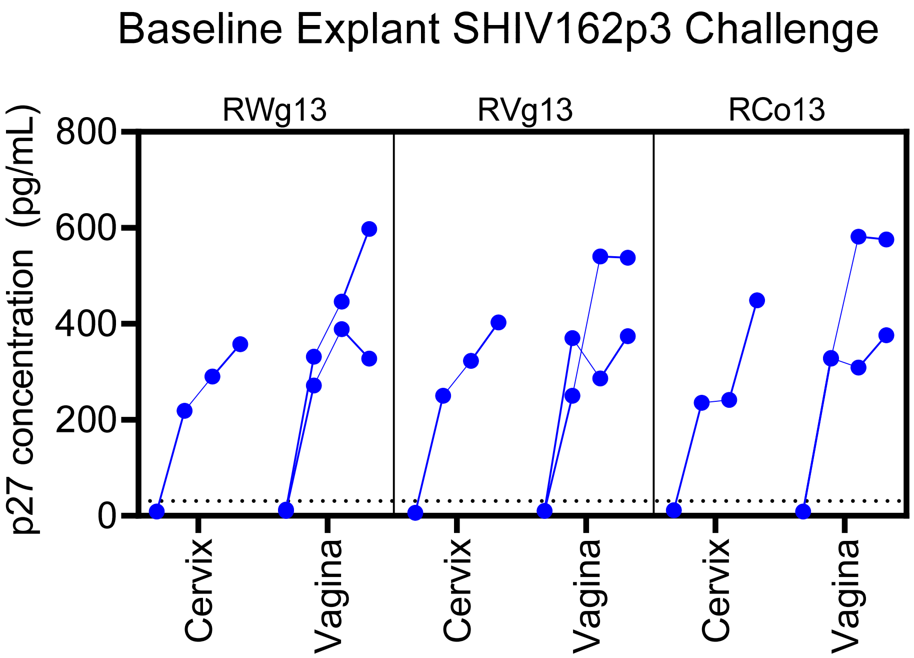

Fig. S5. Baseline vaginal explant challenges. Cervical and vaginal biopsies taken prior to mRNA delivery were challenged with SHIV162p3. Points indicate TZM assays on days 0, 4, 7, and 10 for each biopsy.

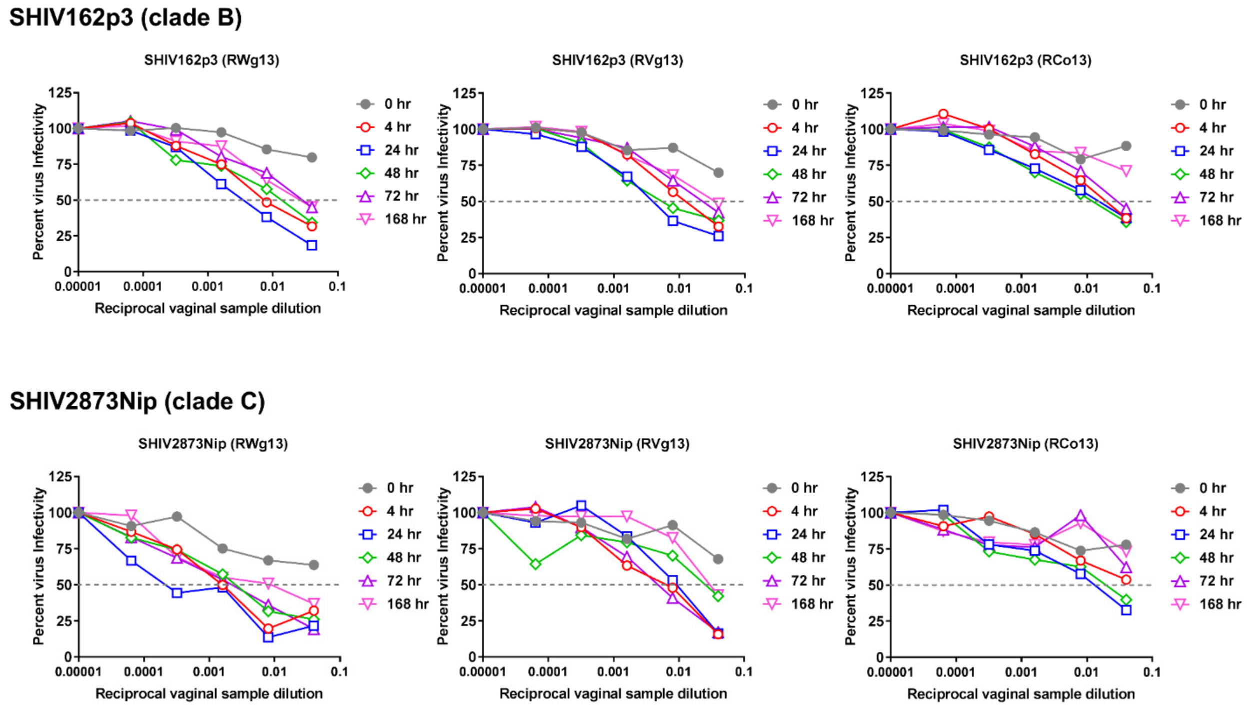

Fig. S6. Vaginal secretion neutralization dilution series. The neutralization activity of genital secretions from animals treated with 250 µg of aPGT121 mRNA at 4 h, 24 h, 48 h, 72 h, and 1-week post-transfection against Clade B and C SHIV strains was evaluated using the *in vitro* TZM-bl assay. Macaque RCo13 was only transfected with aPGT121 heavy chain (i.e. no light chain).

	aPGT121				sPGT121		
	Animal ID				Animal ID		
**Day**	**420**	**456**	**461**	**Mean**	**401**	**414**	**Mean**
**1**	594.0	122.0	299.0	338.3	22.1	100.7	61.4
**7**	60.5	51.6	80.1	64.1	26.1	46.1	36.1
**14**	91.5	68.1	97.7	85.7	13.8	12.8	13.3
**21**	52.2	70.8	149.0	90.6	11.1	8.4	9.8
**28**	35.6	55.2	41.1	44.0			

Table S1: PGT121 concentrations by animal and mean in sheep secretions (µg/mL)

Construct	Sequence
**PGT121 LC-NanoLuc mRNA Geneblock**	CGATTGGCGGAAGGCCGTCAAGGCCGCATTTTTAAGCTTTAATACGAC<br>TCACTATAGGGAAATAAGAGAGAAAAGAAGAGTAAGAAGAAATATA<br>AGAGCCACCATGAAGTGGGTGACCTTCATCAGCCTGCTGTTCCTGTTC<br>AGCAGCGCCTACAGCAGCGACATCAGCGTGGCCCCCGGCGAGACCGC<br>CAGAATCAGCTGCGGCGAGAAGAGCCTGGGCAGCAGAGCCGTGCAGT<br>GGTACCAGCACAGAGCCGGCCAGGCCCCCAGCCTGATCATCTACAAC<br>AACCAGGACAGACCCAGCGGCATCCCCGAGAGATTCAGCGGCAGCCC<br>CGACAGCCCCTTCGGCACCACCGCCACCCTGACCATCACCAGCGTGGA<br>GGCCGGCGACGAGGCCGACTACTACTGCCACATCTGGGACAGCAGAG<br>TGCCCACCAAGTGGGTGTTCGGCGGCGGCACCACCCTCACCGTGCTGA<br>GAGCCGTGGCCGCCCCCAGCGTGTTCATCTTCCCCCCCAGCGAGGACC<br>AGGTGAAGAGCGGCACCGTGAGCGTGGTGTGCCTGCTGAACAACTTC<br>TACCCCAGAGAGGCCAGCGTGAAGTGGAAGGTGGACGGCGTGCTGAA<br>GACCGGCAACAGCCAGGAGAGCGTGACCGAGCAGGACAGCAAGGAC<br>AACACCTACAGCCTGAGCAGCACCCTGACCCTGAGCAGCACCGACTA<br>CCAGAGCCACAACGTGTACGCCTGCGAGGTGACCCACCAGGGCCTGA<br>GCAGCCCCGTGACCAAGAGCTTCAACAGAGGCGAGTGCGGAGGCGGG<br>GGCAGCGTCTTCACACTCGAAGATTTCGTTGGGGACTGGCGACAGAC<br>AGCCGGCTACAACCTGGACCAAGTCCTTGAACAGGGAGGTGTGTCCA<br>GTTTGTTTCAGAATCTCGGGGTGTCCGTAACTCCGATCCAAAGGATTG<br>TCCTGAGCGGTGAAAATGGGCTGAAGATCGACATCCATGTCATCATCC<br>CGTATGAAGGTCTGAGCGGCGACCAAATGGGCCAGATCGAAAAAATT<br>TTTAAGGTGGTGTACCCTGTGGATGATCATCACTTTAAGGTGATCCTG<br>CACTATGGCACACTGGTAATCGACGGGGTTACGCCGAACATGATCGA<br>CTATTTCGGACGGCCGTATGAAGGCATCGCCGTGTTCGACGGCAAAA<br>AGATCACTGTAACAGGGACCCTGTGGAACGGCAACAAAATTATCGAC<br>GAGCGCCTGATCAACCCCGACGGCTCCCTGCTGTTCCGAGTAACCATC<br>AACGGAGTGACCGGCTGGCGGCTGTGCGAACGCATTCTGGCGTGATA<br>AGCTGCCTTCTGCGGGGCTTGCCTTCTGGCCATGCCCTTCTTCTCTCCC<br>TTGCACCTGTACCTCTTGGTCTTTGAATAAAGCCTGAGTAGGAAGGCG<br>GCCGCAAAAACTGGGCC

**DAF GPI anchored PGT121 HC mRNA DNA IVT Template**	TTTTAAGCTTTAATACGACTCACTATAGGGAAATAAGAGAGAAAAGA<br>AGAGTAAGAAGAAATATAAGAGCCACCATGAAATGGGTCACCTTTAT<br>CAGCCTGCTGTTCCTGTTCAGCAGCGCCTACAGCCAGATGCAGCTCCA<br>AGAGTCTGGCCCTGGCCTGGTCAAGCCTAGCGAAACACTGAGCCTGA<br>CCTGTTCCGTGTCCGGCGCCAGCATCAGCGATAGCTACTGGTCCTGGA<br>TCAGAAGAAGCCCCGGCAAAGGCCTGGAATGGATCGGCTACGTGCAC<br>AAGAGCGGCGACACCAACTATAGCCCCAGCCTGAAGTCCAGAGTGAA<br>CCTGAGCCTGGACACCAGCAAGAACCAGGTGTCCCTGTCTCTGGTGGC<br>CGCCACAGCTGCTGATAGCGGCAAGTACTACTGCGCCAGAACACTGC<br>ACGGCAGACGGATCTATGGCATCGTGGCCTTCAACGAGTGGTTCACCT<br>ACTTCTACATGGACGTGTGGGGCAACGGCACCCAAGTGACAGTGTCT<br>AGCGCCAGCACAAAGGGCCCTAGCGTTTTCCCACTGGCTCCCAGCAGC<br>AGAAGCACCAGCGAATCTACAGCCGCTCTGGGCTGCCTCGTGAAGGA<br>CTACTTTCCTGAGCCAGTGACCGTGTCCTGGAACAGCGGCTCTCTGAC<br>ATCTGGCGTGCACACCTTTCCAGCCGTGCTGCAAAGCAGCGGCCTGTA<br>CTCTCTGAGCAGCGTGGTCACAGTGCCTAGCTCTAGCCTGGGCACCCA<br>GACCTACGTGTGCAATGTGAATCACAAGCCCAGCAACACCAAGGTGG<br>ACAAGAGAGTGGAAATCAAGACCTGCGGCGGAGGCAGCAAGCCTCCT<br>ACATGTCCTCCATGTCCTGCTCCAGAAGCTGCCGGCGGACCTTCCGTG<br>TTTCTGTTCCCTCCAAAGCCTAAGGACACCCTGATGATCAGCAGAACC<br>CCTGAAGTGACCTGCGTGGTGGTGGACGTGTCCCAAGAGGACCCCGA<br>CGTGAAGTTCAATTGGTACGTGAACGGCGCCGAGGTGCACCACGCTC<br>AGACAAAGCCAAGAGAGACACAGTACAACAGCACCTACAGAGTGGT<br>GTCCGTGCTGACCGTGACACACCAGGATTGGCTGAACGGCAAAGAGT<br>ACACCTGTAAAGTCTCCAACAAGGCCCTGCCTGCTCCTATCCAGAAAA<br>CCATCAGCAAGGACAAGGGCCAGCCTCGCGAACCCCAGGTTTACACA<br>CTGCCTCCAAGCAGAGAGGAACTGACCAAAAATCAGGTTTCCCTGAC<br>CTGCCTGGTTAAGGGCTTCTACCCCAGCGACATCGTGGTGGAATGGGA<br>GTCTAGCGGACAGCCCGAGAACACCTACAAGACCACACCTCCAGTGC<br>TGGACAGCGACGGCAGCTACTTCCTGTACAGCAAGCTGACAGTGGAC<br>AAGTCCAGATGGCAGCAGGGCAACGTGTTCAGCTGCAGCGTGATGCA<br>CGAGGCCCTGCACAACCACTACACCCAGAAGTCTCTGAGCGTCAGCC<br>CTGGCAAGCACGAGACAACCCCTAACAAAGGCAGCGGCACCACCTCT<br>GGCACCACAAGACTGCTGTCTGGCCACACCTGTTTCACACTGACCGGC<br>CTGCTGGGCACACTGGTTACAATGGGACTGCTGACCTGATAAGCTGCC<br>TTCTGCGGGGCTTGCCTTCTGGCCATGCCCTTCTTCTCTCCCTTGCACC<br>TGTACCTCTTGGTCTTTGAATAAAGCCTGAGTAGGAAGGCGGCCGCAA<br>AAA

Table S2: DNA template sequences used for *in vitro* transcription of mRNA

Primer Name	Purpose	Sequence
**A1: FWD**	**PCR Gibson Overlaps onto PGT121 LC - NanoLuc ORF**	GCGACGATTGGCGGAAGGCCGTCAAGGCCGCATTTTTAAG
**A2: RVS**		CGGGCAGTGAGCGGAAGGCCCATGAGGCCCAGTTTTTG
**B1: FWD**	**Create linear pMA-7 vector template for subsequent Gibson cloning**	GGCCTTCCGCTCACTGCC
**B2: RVS**		GGCCTTCCGCCAATCGTC
**C1: FWD**	**Sanger sequencing of insert fidelity of pMA-7 cloning**	TAAAACGACGGCCAGTGAGCGCGAC
**C2: RVS**		CTGGCACGACAGGTTTCCCGACTGG

Table S3: Primers used during cloning of *in vitro* transcription templates

Movie S1: 3D PET/CT reconstruction of radiolabeled mRNA 24hrs post vaginal aerosol delivery. Macaque RVg13 received 250 µg of ^{64}Cu labelled PGT121 mRNA in two doses, once at the cervix, and another dose 3-4 cm caudally, at the vagina. Contrast levels were set to allow visualization of the relatively weak signal within the draining lymph nodes. Green represents low signal and purple represents high signal. 3D Reconstruction was created using Amira (Thermo Fisher).
