# Supplementary material for: Aerosol Delivery of Synthetic mRNA to Vaginal Mucosa Leads to Durable Expression of Broadly Neutralizing Antibodies against HIV
Source: Mol Ther. 2020 Jan 10;28(3):805–19. doi: 10.1016/j.ymthe.2020.01.002 (PMC7054722; doi:10.1016/j.ymthe.2020.01.002)
Supplement: Document S2. Article plus Supplemental Information [file mmc3.pdf]

Molecular Therapy
Original Article

# Aerosol Delivery of Synthetic mRNA to Vaginal Mucosa Leads to Durable Expression of Broadly Neutralizing Antibodies against HIV

Kevin E. Lindsay,[1,5] Daryll Vanover,[1,5] Merrilee Thoresen,[2] Heath King,[2] Peng Xiao,[3] Peres Badial,[2] Mariluz Araínga,[3] Seong Bin Park,[4] Pooja M. Tiwari,[1] Hannah E. Peck,[1] Emmeline L. Blanchard,[1] Jean M. Feugang,[4] Alicia K. Olivier,[2] Chiara Zurla,[1] Francois Villinger,[3] Amelia R. Woolums,[2] and Philip J. Santangelo[1]

[1]Wallace H. Coulter Department of Biomedical Engineering, Georgia Institute of Technology and Emory University, Atlanta, GA 30332, USA; [2]Department of Pathobiology and Population Medicine, College of Veterinary Medicine, Mississippi State University, Starkville, MS 39762, USA; [3]New Iberia Research Center, University of Louisiana at Lafayette, Lafayette, LA 70560, USA; [4]Department of Animal and Dairy Sciences, Mississippi State University, Starkville, MS 39762, USA

**There is a clear need for low-cost, self-applied, long-lasting approaches to prevent human immunodeficiency virus (HIV) infection in both men and women, even with the advent of pre-exposure prophylaxis (PrEP). Broadly neutralizing antibodies represent an option to improve HIV prophylaxis, but intravenous delivery, cold-chain stability requirements, low cervicovaginal concentrations, and cost may preclude their use. Here, we present an approach to express the anti-GP120 broadly neutralizing antibody PGT121 in the primary site of inoculation, the female reproductive tract, using synthetic mRNA. Expression is achieved through aerosol delivery of unformulated mRNA in water. We demonstrated high levels of antibody expression for over 28 days with a single mRNA administration in the reproductive tract of sheep. In rhesus macaques, neutralizing antibody titers in secretions developed within 4 h and simian-HIV (SHIV) infection of *ex vivo* explants was prevented. Persistence of PGT121 in vaginal secretions and epithelium was achieved through the incorporation of a glycosylphosphatidylinositol (GPI) anchor into the heavy chain of the antibody. Overall, we present a new paradigm to deliver neutralizing antibodies to the female reproductive tract for the prevention of HIV infections.**

## INTRODUCTION

Human immunodeficiency virus (HIV) remains a substantial public health burden worldwide, with 36 million people infected and 1.8 million new cases per year.[1] More than 90% of infections occur via sexual contact and the estimated probability of infection of the female genital tract (FRT) is 1 in 200–2,000 per coital act, depending on the viral burden in the donor.[2,3] Recipient factors providing protection include mucus, antimicrobials present in genital secretions, intact tight junctions between epithelial cells, lactobacillus-dominated microbiome, and genetic factors.[4] The increased use of anti-retroviral therapy (ART) has markedly improved the outlook of HIV-infected patients and the recently approved pre-exposure (PrEP) and post-exposure prophylaxis regimens are >90% efficacious. PrEP however, consists of a daily use regimen, with a high probability of non- or partial adherence, the potential for side effects, expensive cost, and a lack of coverage against other sexually transmitted infections (STIs). Therefore, alternative approaches suitable for self-application in the treatment and prevention of HIV could prove useful.

Virus introduced into the FRT lumen via ejaculate or released from infected donor cells rapidly permeates the vaginocervical epithelium by passive diffusion, perhaps as quickly as within 30 min[5,6] and reaches systemic lymph nodes in less than 24 h.[7] Thus, intervention strategies should counteract initial virus seeding and replication in the lower FRT and prevent trafficking of virions to regional lymph nodes.

HIV envelope (*Env*) is expressed on the surface of virions and infects cells as a trimeric glycoprotein of two non-covalently associated subunits, gp120 and gp41, responsible for binding CD4 receptors on target cells and mediating membrane fusion, respectively. For genital immunoglobulin G (IgG), it appears that neutralization of *Env* is a major factor in protection from viral acquisition.[8]

Broadly neutralizing antibodies (bNAbs), characterized by their ability to neutralize a broad assortment of HIV strains with high potencies, have been isolated from a small subset of infected individuals.[9] Passive immunoprophylaxis by parenteral administration of bNAbs has been shown to prevent infection,[10–12] and even post-exposure,[13] and is currently being tested

Received 18 November 2019; accepted 14 December 2019;
https://doi.org/10.1016/j.ymthe.2020.01.002.

[5]These authors contributed equally to this work.

**Correspondence:** Philip J. Santangelo, Wallace H. Coulter Department of Biomedical Engineering, Georgia Institute of Technology and Emory University, Atlanta, GA 30332, USA.
**E-mail:** philip.santangelo@bme.gatech.edu


This is an open access article under the CC BY license (http://creativecommons.org/licenses/by/4.0/).

for its ability to reduce viral reservoirs in HIV-infected patients[14,15] and suppress viral recrudescence after ART interruption.[16] In one study, administration of bNAbs to monkeys during acute SHIV infection led to long-lasting $CD8^+$ T cell immunity that suppressed virus long after administered antibody titers were undetectable.[17]

To protect patients from mucosal HIV infection, antibodies delivered parenterally must reach the genital compartment through transudation from the serum. It has been estimated that the concentration of antibody in the serum is approximately 90-fold higher than in vaginal secretions.[2] The amount of bNAb required in genital secretions to prevent infection decreases with higher binding affinities. For the bNAb PGT121, with a relatively high binding affinity ($K_D$ = 0.086 nM), genital secretion concentrations as low as 30 ng/mL are estimated to provide neutralizing protection.[2] There also appears to be a time delay between the peak serum concentration of injected antibody (which is almost immediately) and the peak concentration in the vaginal secretions, although the magnitude of this delay is dependent on the specific antibody.[12,18] While systemically administered bNAbs have demonstrated promising anti-viral properties, the large doses and uncertain time required for genital secretions and tissues to reach sufficient concentrations suggest that local application of bNAbs could provide a viable alternative. Direct vaginal application of bNAbs before challenge protected non-human primates (NHPs),[19] but the half-life of such an approach is likely on the order of several hours.[20]

Our group recently demonstrated that administration of mRNA in water by lung aerosol led to the expression of membrane-anchored neutralizing antibodies against respiratory syncytial virus that protected mice from infection.[21] Here, we explored the use of the cervicovaginal mucosa as a platform to express mRNA-encoded bNAbs at the site of infection. Specifically, we demonstrated that aerosolized mRNA induces expression of PGT121 at high concentrations in the vagina and cervix in sheep and rhesus macaques. These models were chosen because (1) sheep are a reasonably accurate anatomical model of the adult human FRT and (2) macaques are compatible with SHIV infections, allowing for neutralization assays in cervical biopsies and secretions.[22–25]

Critically, protein expression was detected within 4 h post-delivery of mRNA. Incorporation of a glycosylphosphatidylinositol (GPI) membrane anchor to the heavy-chain (HC) Fc domain of PGT121 (aPGT121), allowed for protein expression up to 28 days after a single administration. In comparison, secreted PGT121 levels decreased substantially by 3 weeks. Furthermore, in rhesus macaques, aPGT121 was able to inhibit SHIV infections of cervicovaginal biopsies infected *ex vivo* as a challenge model.[26] Overall, we present a new paradigm for transient expression of neutralizing antibodies against HIV in the female genital tract, with the possibility of multiplexing against other pathogens.

# RESULTS

## Aerosolized Transfection Delivers mRNA into the Cell Cytoplasm

We hypothesized, based on our prior work, that aerosolizing mRNA diluted in water may transfect other mucosal interfaces, such as the FRT.[21] This method avoids induction of innate immunity and inflammation, which can be activated by many common synthetic mRNA carriers,[27,28] and facilitates potential translation to the clinic. The use of water as an mRNA solvent is substantiated by prior research, which demonstrated that hypotonic formulations markedly increased the rate at which small molecule drugs and muco-inert nanoparticles reached the vaginal epithelial surface in mice[29,30] or rectal epithelial tissues in monkeys.[26] We employed a vertical *in vitro* apparatus to transfect cell monolayers with aerosolizers (Figure S1A). Upon aerosol transfection to Vero cells, the mean fluorescence intensity (MFI) of mRNA-encoded GFP was significantly higher compared to either the pDNA-encoded or control treatments (Figures 1A and 1B).

To initially investigate the uptake mechanism involved with aerosol-based transfection, we transfected monolayers of adherent cells with Dylight 650 fluorescently labeled GFP mRNA, through the use of complementary oligos annealed to its 3′ untranslated region (3′ UTR).[31–33] At discrete time points after spraying, the fraction of nucleic acid outside of the endosomal compartment or nucleus was estimated using fluorescence microscopy (Figure 1C). Since approximately 30% of the mRNA signal overlapped with endosomal markers, it follows that approximately 70% of aerosolized GFP mRNA was found to be cytosolic 15 min post-delivery (Figure 1D). There was a slight, but not significant, increase in endosome localization over time (up to 6 h) (Figure 1E). Therefore, aerosol transfection likely delivers unformulated nucleic acids directly into the cytosol of cells, allowing rapid access to the ribosomal machinery and subsequent protein expression.

## mRNA Aerosolization Is Required to Transfect the Cervix Epithelium

We explored the spatiotemporal parameters governing FRT transfection in sheep, which have geometrically similar FRT compared to humans. In the sheep used in this study, the distance from the vaginal vestibule to the cervix was 9 –11 inches. We delivered 250 μg of firefly luciferase encoding mRNA in water to the FRT via a MADgic Teleflex aerosolizer (Figures S1B and S1C). After 24 h, the entire FRT was removed during postmortem necropsy, D-luciferin substrate was applied, and an *In Vivo* Imaging System (IVIS) was used to record the bioluminescence signal. As an alternative delivery method, mRNA was “squirted” by high-pressure syringe onto the cervix. Robust transfection of the cervix was observed by aerosolization, but no signal was detected using a high-pressure syringe (Figure 1F). These data indicate that aerosolization is a reliable means to transfect the cervix epithelium with synthetic mRNA in water.

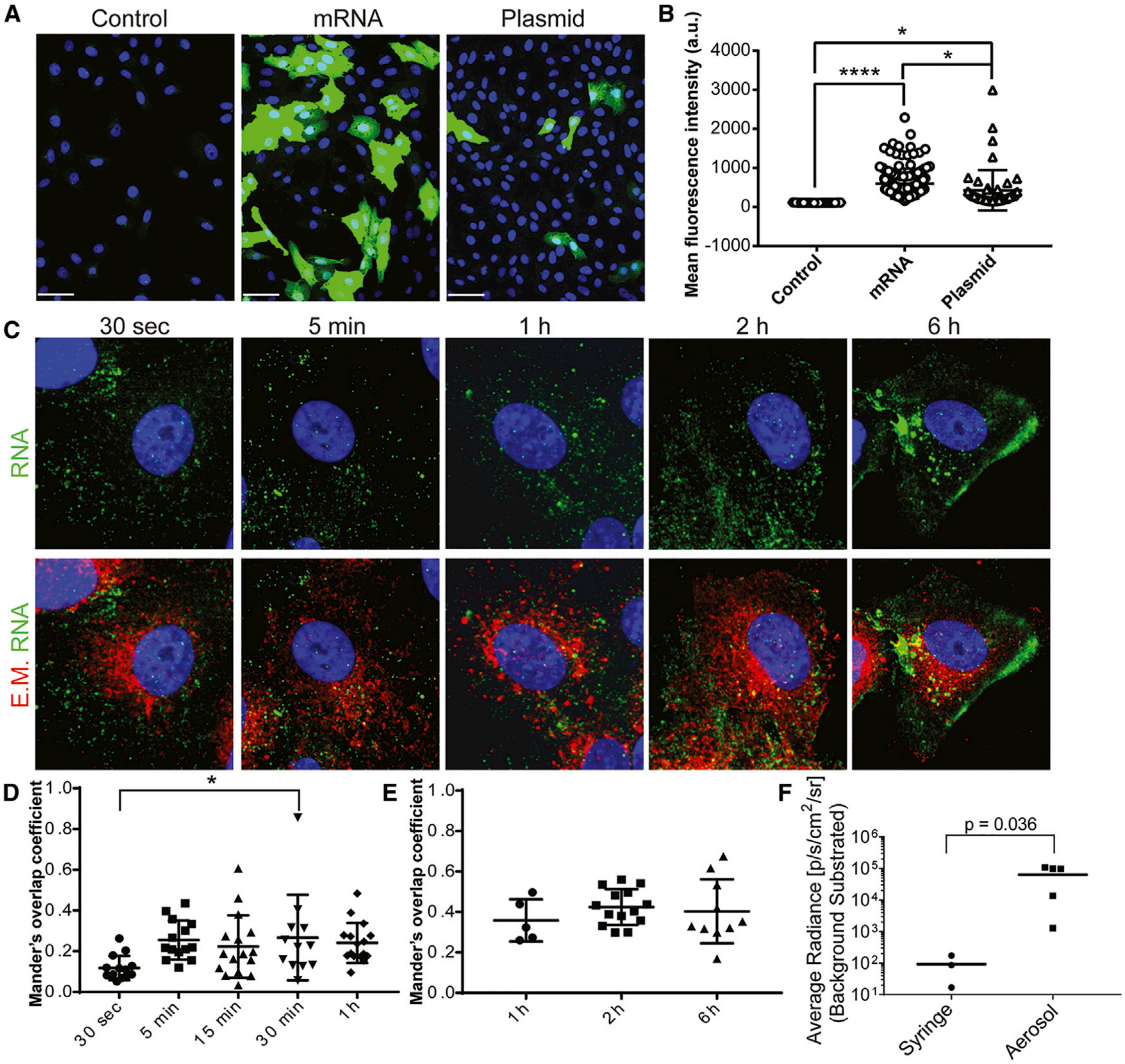


**Figure 1. Aerosolized mRNA in Water Transfects Vero Cells and Ovine Cervical Epithelium, Likely through Direct Cytosolic Entry**

(A) Vero cells were transfected via aerosol with $H_2O$ (control), GFP-encoding mRNA, or GFP-encoding plasmid complexed with Lipofectamine 2000 (pDNA$^+$L2k). Cells were fixed and imaged for GFP (green) at 24 h. Scale bars represent 30 μm. (B) Quantification of the GFP MFI on a per-cell basis, for each transfection condition. (C) Dye-labeled mRNA (green) was delivered via aerosol to Vero cells and fixed at 30 s, 5 min, 1 h, 2 h, and 6 h post-transfection. Cells were then stained with a pan-endosomal marker cocktail including: CD63, clathrin, caveolin, EEA1, and LAMP-1 (red). Scale bars represent 10 μm. (D) The extent of spatial overlap between the endosomal markers and mRNA at each time point, up to 1 h. The Mander's overlap coefficient reflects the proportion of mRNA that overlaps with the endosomal markers. (E) The proportion of mRNA overlapping with the endosomal markers from 1 h to 6 h; contrast enhanced on a different scale than the acute time points in (D). (F) In live sheep, 250 μg of firefly luciferase mRNA in $H_2O$ was delivered to the cervix as a single dose with either a high-pressure syringe or via aerosolizer. A speculum was used to visualize the cervix in sedated sheep during transfection. 24 h post-transfection, the animals were euthanized and subjected to necropsy. Luciferin was added to isolated cervix and luminescence quantified as the average radiance (p/s/cm$^2$/sr) per animal via an IVIS Lumina imaging system. Statistical comparison was performed using two tailed Mann-Whitney non-parametric analyses. For all panels, *$p < 0.05$, ****$p < 0.00005$; error bars represent ± 95% confidence interval (CI).

### PGT121-NanoLuc Anchored to the Membrane of Cells Binds SHIV

To ensure that experimentally relevant epithelium tissue was examined during necropsy and subsequent tissue processing, a luminescence reporter, nanoluciferase (NanoLuc), was fused to the 3′ end of PGT121 light chain (LC) (Figure 2A). Secreted PGT121-NanoLuc (sPGT121-NanoLuc) was isolated by gentle protein A/G purification of the supernatant after transient transfection of adherent cells. The dynamic range for PGT121-NanoLuc in solution was found to be nearly six orders of magnitude (Figure S2). In a modified gp120 ELISA assay a sensitivity of approximately 500 pg/mL was observed, on par with conventional ELISA readouts, but with a greater dynamic range (Figure S2).

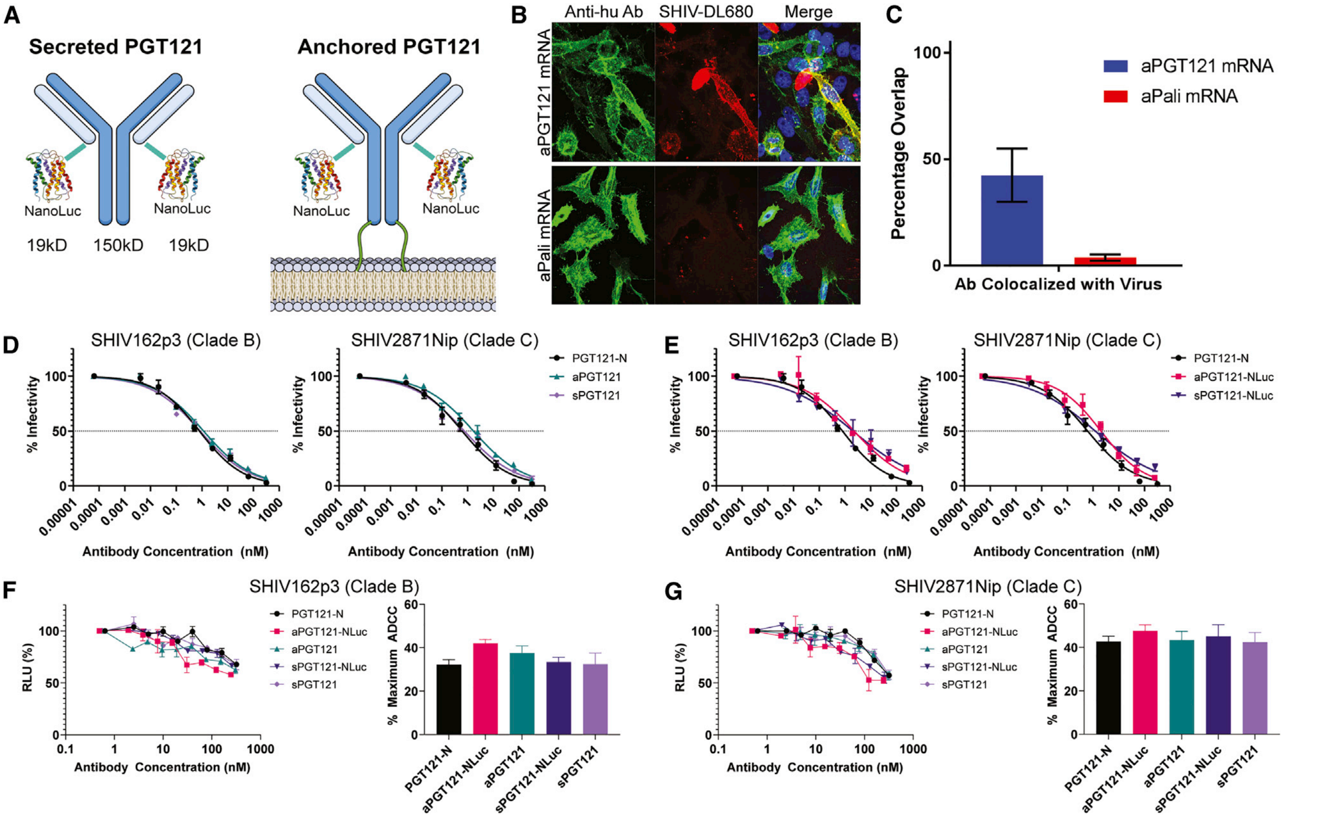


**Figure 2. Transfected Cells Displaying Membrane-Anchored PGT121 on Their Surface Bind SHIV Virions**

(A) Schematic of the PGT121-NanoLuc fusion protein, in secreted and anchored forms. NanoLuc was fused to the 3′ end of the kappa light chains (2 per antibody molecule). In the case of the membrane-anchored PGT121, a GPI anchor was fused to the 3′ end of the HC. Total MW of anchored PGT121-NanoLuc was 195 kDa (B) HEK293 cells were transfected with 1 μg of PGT121 mRNA, delivered at a 4:1 ratio of HC to LC with Lipofectamine 2000 (top row). Respiratory syncytial virus neutralizing anchored Palivizumab (aPali) antibody mRNA was used as a control comparison (bottom row). 24 h later, DyLight 680-labeled SHIV particles (red) were incubated with the transfected cells for 4 h without agitation. After washing 3×, the cells were fixed and immunostained with an anti-human secondary antibody (green). (C) Average percentage overlap between SHIV virus and anchored antibodies from panel (B), using 30 cells per condition. The error bars represent the mean ± 95% CI. (D) Anchored and secreted PGT121 was produced in Vero cells and purified. The mRNA-expressed antibodies were compared against parental PGT121 for SHIV neutralization with either a clade B or clade C SHIV isolate. Error bars represent SD. (E) Anchored and secreted PGT121 fused to NanoLuc was produced in Vero cells and purified. The mRNA-expressed NanoLuc fusion antibodies were compared against parental PGT121 for SHIV neutralization with either a clade B or clade C SHIV isolate. Error bars represent SD. (F) mRNA-expressed anchored or secreted PGT121 either with or without NanoLuc was purified, diluted, and tested for ADCC against SHIV162p3 (clade B). Complete dilution series (left) and percent maximal ADCC (right) are shown. Error bars represent SD. Percent maximum ADCC compared by Kruskal-Wallis. (G) The same antibodies from (F) were purified, diluted, and tested for ADCC against SHIV2871Nip (clade C). Complete dilution series (left) and percent maximal ADCC (right) are shown. Error bars represent SD. Percent maximum ADCC compared by Kruskal-Wallis.

Additionally, the GPI anchor of decay-associated factor (DAF) was fused to the 3′ end of the HC domain of the PGT121 mRNA transcript (Figure 2A).[34,35] The complete IgG, anchored PGT121 (aPGT121), efficiently localized to the cell surface when GPI anchored-HC and LC mRNA transcripts were delivered simultaneously at a 1:4 mass ratio (Figure S3). As expected, the delivery of mRNA encoding either single chain alone did not result in significant staining with an anti-human secondary antibody, indicating incomplete antibody formation.

Expressed aPGT121 retained the ability to bind SHIV virions when displayed on the cell membrane. HEK293 cells were transiently transfected with either aPGT121 or anchored palivizumab (aPali) mRNA 24 h prior to addition of fluorescently labeled SHIV AD8EO virions, and the cells were analyzed by confocal microscopy (Figure 2B). Colocalization analysis indicated that aPGT121-expressing cells, but not aPali, were able to bind SHIV virions and capture them at the surface of transfected cells (Figure 2C).

We then purified mRNA-expressed aPGT121 and sPGT121 with and without a NanoLuc and compared the clades B (SHIV162p3) and C (SHIV2871Nip) SHIV-neutralizing capacity of each construct to a parental PGT121 produced in *Nicotiana* (PGT121-N). While both the anchored and secreted forms of

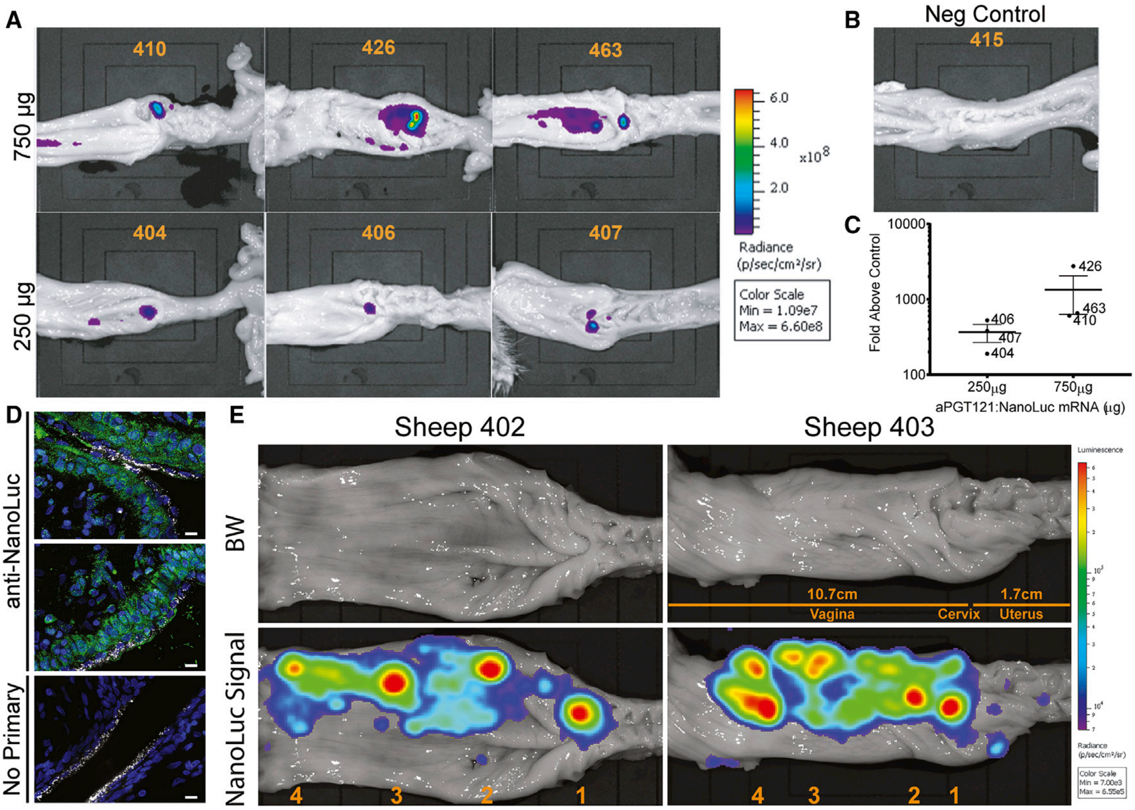


**Figure 3. The Entire Sheep Cervicovaginal Epithelium Is Receptive to Transfection by Large Doses of aPGT121 mRNA**

(A) A single 250 μg or 750 μg dose of aPGT121-NanoLuc was delivered by aerosol to the cervix. After 24 h, sheep were euthanized, FRT excised, luciferin added, and luminescence measured via IVIS. Representative images demonstrating the intensity in each animal are displayed. (B) The FRT from an untreated sheep processed in the same manner as in (A). (C) The average radiance at the cervix over the average radiance in the control animal, for each mRNA dosing group. Error bars represent the ± 95% CI. (D) In 2 animals, aerosolized dye-labeled mRNA was delivered as previously described. After 24 h, the cervix was excised and processed for immunofluorescence tissue imaging. The bottom panel represents secondary only control. DAPI, cell nuclei; white, aPGT121 mRNA; green, anti-NanoLuc antibody. Section scale bars represent 10 μm. (E) aPGT121-NanoLuc mRNA was sprayed in four consecutive 750 μg doses, beginning at the cervix (marked as "1") and retracting caudally to distal vagina ("4").

mRNA-expressed PGT121 without a NanoLuc were not significantly different from PGT121-N (Figure 2D), the fusion of the NanoLuc reporter had a minor effect on the neutralization of both clades (Figure 2E). Hence, aPGT121-NanoLuc anchored to the plasma membrane retained *Env* binding capacity and neutralizing capacity.

Finally, to verify that mRNA-expressed antibodies retained similar Fc function to the parental PGT121, we conducted an antibody-dependent cell-mediated cytotoxicity (ADCC) assay using the same clade B and clade C SHIV strains, respectively. No detectable difference was measured between any of the mRNA-expressed antibody forms and PGT121-N with either viral clade (Figures 2F and 2G). Notably, the concentration of PGT121 required to elicit 50% neutralization or ADCC are significantly different. This is consistent with previous reports that neutralizing capacity and effector cell function, while related, are not completely dependent on one another, and the relationship between titers for each assay can vary depending on virus strain and neutralizing antibody used.[36] Therefore, mRNA-expressed antibodies, regardless of membrane-anchor or NanoLuc modification, retain neutralizing capacity and effector cell function comparable to the commercial PGT121.

### The Entirety of the Lower FRT Can Be Transfected by mRNA Aerosol

In the luciferase expression experiments in Figure 1, a dose of 250 μg mRNA was arbitrarily chosen. To determine whether higher doses of mRNA lead to production of more antibody, we treated three sheep each with either 250 μg or 750 μg of mRNA; all treatments were administered in a 300 μL volume. After 24 h, the FRT was removed after euthanasia and necropsy and 2 mL of NanoLuc substrate was applied to cover the entire vaginal, cervical, and uterine epithelium; IVIS imaging was used to observe the location and quantify the luminescence from each animal (Figure 3). Luminescence at and surrounding the cervix was higher in all 750 μg dosed animals (Figures

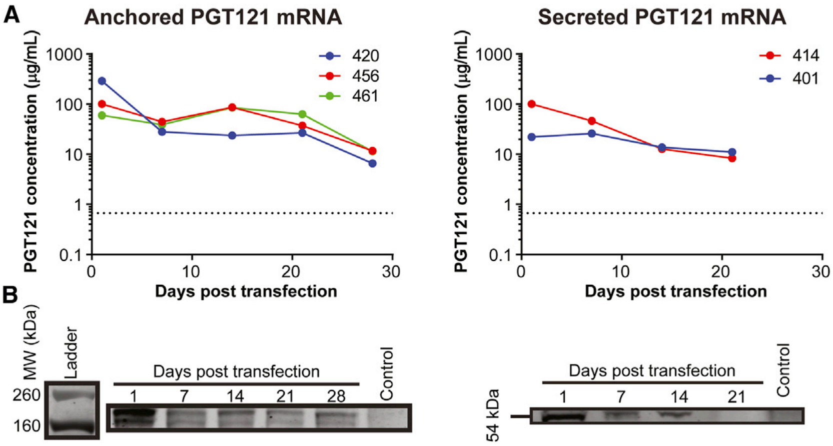


**Figure 4. aPGT121 Persists in Vaginal Secretions out to 28 Days Post-transfection**

Three sheep were transfected with two doses of 750 µg of mRNA encoding for aPGT121, while another two sheep were transfected with an equal mass of sPGT121 mRNA. Vaginal secretions were collected at 1, 7, 14, 21, and 28 days post-transfection. (A) Longitudinal sampling of PGT121 concentrations over time, for all animals. The horizontal asymptote represents the limit of detection. Individual and mean values can be found in Table S1. (B) Antibody concentrations in vaginal secretions were quantified via western blot analysis, using a standard curve of purified PGT121-NanoLuc protein. The expected size of aPGT121-NanoLuc was confirmed to be around 195 kDa. For secreted antibody, the LC uncoupled from the HC, resulting in a 54 kDa band.

3A–3C). Given the increase in expression, subsequent experiments utilized 750 µg of mRNA per dose.

To visualize mucosal transfection via microscopy, we delivered fluorescently labeled mRNA to the cervix via aerosol. At 24 h, FRT tissue was removed, fixed, and immunostained for NanoLuc, allowing for both mRNA and protein visualization. Expressed aPGT121 was localized to both the cervical epithelial layer and stromal cells (Figure 3D). Surprisingly, both mRNA and protein expression were detected well below the outer-most epithelial layers. A secondary only control demonstrated a lack of non-specific binding by the secondary antibody.

We next explored whether both the vaginal and cervical epithelia were equally permissive to transfection. Four sprays, 750 µg each, were applied sequentially starting at the cervix, with 2–3 cm retractions after each administration (Figure 3E). The amount and distribution of expressed protein were assessed via IVIS imaging at 24 h. In both tested animals, aPGT121-NanoLuc expression was detected throughout the vagina and cervix with similar luminescence intensity maximums (Figure 3E). These data suggest that both the vagina and cervix epithelia can be efficiently transfected via aerosol.

### Membrane Anchoring Retains PGT121 in Genital Secretions and FRT Epithelium

Our next goal was to evaluate the pharmacokinetics of the anchored (aPGT121) and secreted (sPGT121) forms of PGT121 over 1 month in two physiologically relevant compartments—genital secretions and the FRT mucosa. We hypothesized that aPGT121 would be retained in the secretions and mucosa longer than sPGT121. After delivery of two 750 µg doses of either aPGT121 or sPGT121 to sheep, genital secretions were collected longitudinally on a weekly basis and PGT121 concentrations were quantified via western blot (Figure 4; Figure S4). The GPI anchor retained aPGT121 at high concentrations in genital secretions compared to sPGT121. Secretion kinetic patterns of aPGT121 were consistent: maximum bNAb concentration at 24 h, followed by a plateau from 7 to 21 days, after which concentrations began to decrease (Figures 4A and 4B; Table S1). At 28 days, the average aPGT121 concentration was 40 µg/mL. In contrast, sPGT121 concentrations peaked at 24 h and then decreased to barely above 10 µg/mL at 14 days. The maximum mean concentration of PGT121 was achieved at 24 h, with 210 µg/mL and 80 µg/mL for anchored and secreted PGT121, respectively. Since we did not assay sPGT121-treated animals at 28 days, it is possible that anchored and secreted antibody concentrations are similar at this time point.

We also evaluated the distribution of PGT121 on the mucosal surface over time. Anchoring PGT121 to the cell membrane surface retained the expressed antibody in the epithelium for at least 1-month post-transfection, while sPGT121 amounts were significantly reduced by 2 weeks (Figure 5). PGT121-NanoLuc in the mucosal compartment was evaluated at 14 and 28 days post-transfection in two ways: (1) IVIS imaging of NanoLuc signal directly following necropsy (Figures 5A and 5B) and (2) quantitative western blot[37] following tissue pulverization (Figures 5C–5E). The luminescence signal in all aPGT121 tissues ($n = 6$) was higher than in sPGT121 tissues ($p = 0.024$). sPGT121 transfected animals at 28 days were originally scheduled but were repurposed, after assessing the low signal results at 14 days. Again, because we did not assay sPGT121-treated animals at 28 days, it is possible that anchored and secreted antibody concentrations are similar at this time point, and such studies should be performed in future work.

From each FRT explant taken at 28 days, regions of caudal vagina, rostral vagina, cervix, and uterus were removed, using the IVIS signal as a guide (Figure 5C). We estimated by western blot that the average concentration of aPGT121 across the three animals was above 1 µg per mg of tissue (Figure 5D). Surprisingly, similar quantities of antibody were measured in each section of the FRT, for a given animal (Figures 5D and 5E). Interestingly, while only rostral vagina and cervix received doses of mRNA, aPGT121-NanoLuc localized to other regions of the FRT, likely a result of DAF GPI linker re-association with cellular membranes after release.[38]

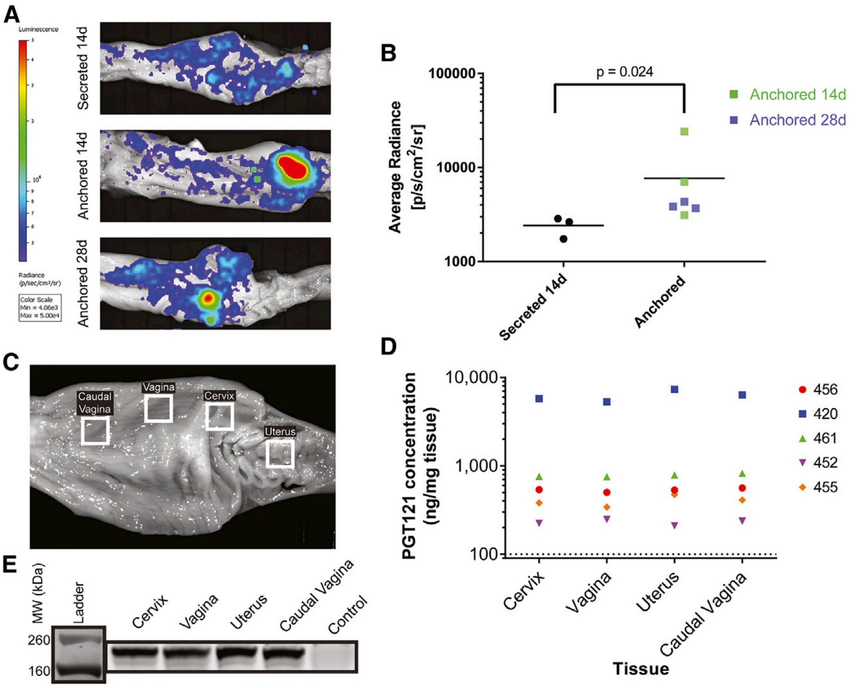


**Figure 5. aPGT121 Persists in the FRT Mucosa at 28 Days Post-transfection**

(A) IVIS imaging of the excised lower FRT at 14 days and 28 days post-transfection. 28-day samples for sPGT121 transfected animals were not collected due to the low signal observed at 14 days. (B) Average radiance of the secreted and anchored PGT121-NanoLuc constructs. Mann-Whitney non-parametric analysis was used to compare the two groups (C) After 28 days, tissue samples were excised under the guidance of IVIS signal, snap-frozen, and pulverized for downstream western blot analysis. (D) Day 28 post-transfection aPGT121 concentrations in excised cervix, vagina, uterus, and caudal vagina were estimated using quantitative western blot. Five animals, euthanized at 28 days, were used in total. (E) Western blot demonstrating the characteristic aPGT121 band at 195 kDa for all regions of the FRT of one treated sheep, compared to control cervix.

### PET/CT Imaging Detects mRNA in the FRT

To characterize aerosolized mucosal mRNA trafficking and localization, we labeled PGT121 encoding mRNA orthogonally with $^{64}$Cu, an approach described recently by our group.[39] 250 μg of radiolabeled mRNA was delivered in two 125 μg doses, one at the ectocervix and a second dose after a 2–3 cm retraction of the atomizer within the vagina. Three rhesus macaques were treated in total. PET/CT imaging was performed at 70 min, 4 h, 24 h, 48 h, and 72 h post-administration (Figure 6).

Our visualization target during speculum insertion and mRNA delivery was the cervical os, which was the focal point of mRNA transfection, as resolved in the PET/CT imaging (Figure 6A; Video S1). Between 70 min and 4 h, the PET signal in the FRT decreased by approximately 50%–67% (Figure 6B). After this initial rapid decrease, the rate of standard uptake value (SUV) decline from 4 h to 72 h decreased and resembled a more linear process (Figure 6B). A potential cause of the rapid decrease over the first 4 h is that excess mRNA in the delivery volume was excreted from the vagina. Excess mRNA is not surprising considering that the original aerosol parameters were optimized in sheep, and the vaginal vault of a rhesus macaque is smaller; therefore, aerosol parameters were adjusted for the remainder of the NHP studies. Hence, the radioactive signal also provided useful insight into the efficiency of the delivery method.

When the amount of radioactivity in the FRT ($SUV_{FRT}$) was reported relative to the total radioactivity in the body ($SUV_{Whole\ Body}$), then the relative amount of mRNA within the FRT remained constant over 48 h and increased between 48 to 72 h (Figure 6C). This relative increase in mRNA concentration in the FRT is likely due to the SUV values in other organs, mainly draining lymph nodes (LNs) (Figure 6D), decreasing below the limit of detection due to either mRNA or radioactive decay.

A large portion of the mRNA (>99%) was restricted to the FRT, while a small fraction of the mRNA was also detected via PET/CT in LNs 70 min after aerosolized delivery (Figure 6D), including sacral, popliteal, inguinal, femoral, iliac, para-aortic, and mesenteric LNs. After 48 h, the signal in the LNs was no longer detectable. However, signal was still abundant in the FRT mucosal surface after 72 h (Figures 6A and 6C), indicating retention of mRNA within transfected cells.

### Cervicovaginal Biopsy Explants Are Protected from SHIV Challenge

To test protective efficacy across the FRT, we used an *ex vivo* biopsy challenge model. Four female rhesus macaques were used: two were sprayed with 250 μg, one with 400 μg, and one with 1,000 μg of aPGT121 mRNA. To minimize potential leakage of excess mRNA solution, we reduced the dose volume to 150 μL. mRNA was delivered in two sequential doses, once at the ectocervix and then another dose 2–3 cm rostral in the vagina. Biopsies were collected before and at 1 day post-transfection. In the 250 μg group, one sample was taken from the endocervix and two from the vagina, while in the 400 and 1,000 μg groups, two samples each were taken from the uterus, endocervix, ectocervix, proximal vagina, and distal vagina. FRT biopsies were then challenged with SHIV162p3, a clade B *Env* virus. We observed a dose-dependent decrease in p27 production in the biopsy explants, with all baseline biopsies exhibiting productive infection (Figure 7A; Figure S5). While one explant produced strong p27 at the 250 μg dose, this effect was diminished at 400 μg dose and completely abrogated at the 1,000 μg dose, where none of the 10 biopsies from 5 different regions of the FRT yielded productive SHIV infection.

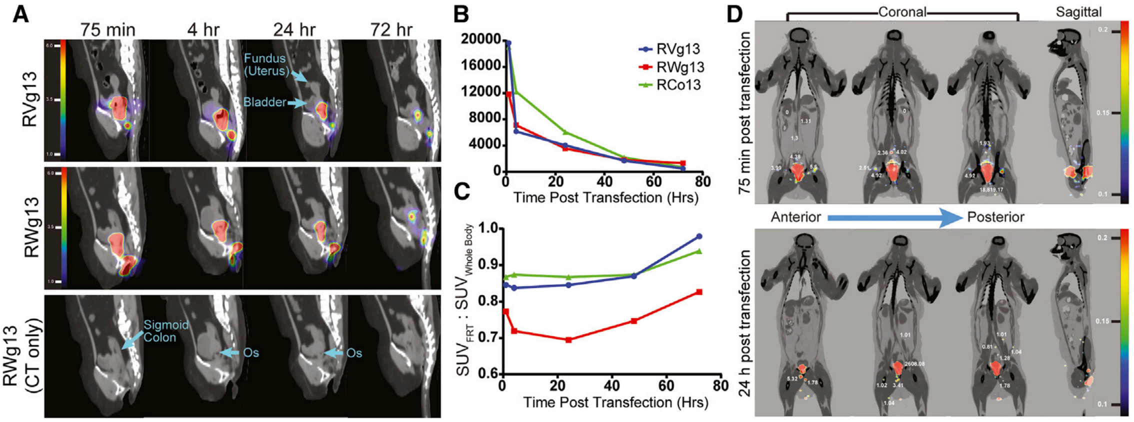


**Figure 6. Longitudinal PET/CT Monitoring of $^{64}$Cu Radiolabeled aPGT121 mRNA after Aerosolized FRT Delivery**

(A–C) Two 125 μg doses of $^{64}$Cu radiolabeled aPGT121 mRNA were delivered via aerosol to first the cervix, then ~3–4 cm caudally in the vagina. A total of 200 μCi of $^{64}$Cu was administered. PET/CT imaging over 3 days was used to monitor mRNA biodistribution. (A) Representative PET/CT images of the abdomen and pelvis from 70 min to 72 h. Contrast enhancement was adjusted to reflect the high SUV signals within the FRT. (B) The total SUV in the FRT over time. (C) The ratio of the total SUV in the FRT to the total SUV contained within the entire body. (D) Whole-body PET/CT images of macaque RVg13 at 70 min and 24 h post-transfection. Numbers in white represent the total SUV within the nearby organ. Contrast enhancement was adjusted to allow visualization of draining lymph nodes.

### *Genital Secretions from aPGT121 mRNA-Treated Macaques Neutralize SHIV* In Vitro

To examine the functional activity of aPGT121, we collected secretions from three biopsied macaques prior to transfection and post-transfection with a 250 μg dose of aPGT121 mRNA at 4 h, 24 h, 48 h, 72 h, and 7 days. It is important to note that macaque RCo13 was transfected with an equal mass of only HC of aPGT121 mRNA. The secretions were evaluated for their ability to neutralize SHIV through the TZM-bl neutralization assay (Figure 7B; Figure S6). Genital secretions from animals transfected with whole aPGT121 mRNA demonstrated an ability to neutralize SHIV162p3 (clade B) and SHIV2873Nip (clade C)[40] out to 1 week. Critically, by 4 h post-transfection, genital secretions already displayed substantial neutralizing activity. The kinetics over time for the HC only (macaque RCo13) construct suggest that without the association of the constant regions of the HC and LC, either the binding pocket is compromised, resulting in reduced neutralization, or ER trafficking is modulated by binding of the ER molecular chaperone HC binding protein (BiP).[41]

## DISCUSSION

The vaginal mucosa has been explored to deliver drugs both locally and systemically due to its large surface area, a high degree of vascularization, avoidance of first-pass metabolism by the liver, good drug permeability, and its accessibility to allow self-application.[42] Though several approaches have had successes, protection from HIV acquisition has been a challenge, because most antimicrobials also damage the epithelium, creating inflammatory conditions and a portal of entry for the virus.[43,44]

With the aerosolized, unformulated mRNA transfection platform described here, the local production of transgene protein by the native epithelium ensures that high tissue and secretion concentrations are achieved quickly, while also ensuring that host glycosylation and other post-translational motifs are preserved. The entire lower FRT was conducive to transfection by aerosolized, naked, synthetic mRNA. Tethering PGT121 to the transfected cell surface using a GPI anchor promoted genital secretion and tissue concentrations well above neutralizing concentrations at 28 days post-transfection. Moreover, mRNA-expressed aPGT121 retained the neutralizing capacity and effector-cell function of parental PGT121. *Ex vivo* challenge of rhesus macaque FRT biopsy explants with SHIV demonstrated tissue level protection due to aPGT121 tissue expression, as well as antibody neutralization activity in secretions.

Recent data using fluorescent HIV suggest that all areas of the female reproductive tract can be considered vulnerable to infection.[5] Thus, prophylactic approaches will likely need to protect the entirety of the lower FRT. IVIS imaging at 1, 14, and 28 days demonstrated that the entirety of the lower FRT epithelium expressed aPGT121. However, by 14 and 28 days, the cervix was the site of the majority of luminescent signal, perhaps in part due to differential turnover and shedding of epithelial cells, differences in cellular metabolism, and the more superficial nature of basal stem cells at the cervix compared to the stratified vagina. Surprisingly, at 28 days post-transfection, tissue lysates from each portion of the FRT (caudal vagina, rostral vagina, cervix, and uterus) still contained substantial aPGT121 concentrations, even though only rostral vagina and cervix were directly transfected by aerosol. Taking into account that the GPI membrane anchor is not a permanent tether and proteins can be cleaved by endogenous glycolipidases,[45] it is possible that GPI anchored PGT121 is able to associate with cell membranes of non-transfected cells, as suggested by prior studies.[46,47] In the case of genital secretions, the source of aPGT121 is likely a combination of enzyme GPI anchor cleavage and cell membranes, whether in the form of non-adhered cells or as fragments, such as exosomes.

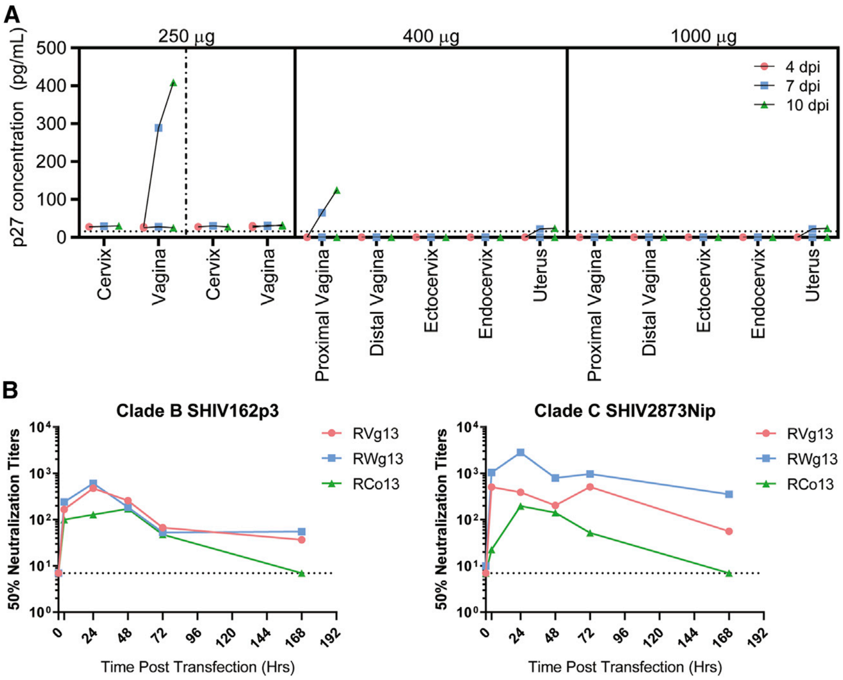


**Figure 7. aPGT121 mRNA Protects Rhesus Macaque Biopsy Explants from SHIV162p3 Challenge and Is Neutralizing in Genital Secretions**

(A) 250 (n = 2), 400 (n = 1), or 1,000 µg (n = 1) of aerosolized aPGT121 mRNA was delivered to the ectocervix and rostral vagina of rhesus macaques. From the 250 µg group, biopsy explants were collected from the endocervix and vagina at 24 h post-transfection, while from the 400 and 1,000 µg groups, two biopsies were collected from each of the indicated regions at 24 h post transfection. Dotted vertical line separates the two animals in the 250 µg group. Biopsies were then challenged with SHIV162p3 virus. Baseline biopsy challenge data can be found in Figure S5. (B) The neutralization activity of genital secretions at 4 h, 24 h, 48 h, 72 h, and 1-week post-transfection against clade B and C SHIV strains was evaluated using the *in vitro* TZM-bl assay. All macaques were treated with 250 µg of aPGT121 mRNA. Macaque RCo13 was only transfected with aPGT121 HC (i.e., no LC). The dashed horizontal line represents the lower limit of detection. Complete dilution series neutralization data can be found in Figure S6.

In this study, the average PGT121 concentrations in both genital secretions and mucosal epithelium remained above the predicted $IC_{50}$ concentration of 30 ng/mL at 30 days.[2] The amount of PGT121 required in the cervicovaginal mucosa (as opposed to the amount within the secretions) to provide adequate protection is unknown. Ultimately, *in vivo*, both compartments will contribute to protecting against HIV infection as virus is introduced into the vaginal lumen and diffuses into the mucosa in search of underlying target cells. Passive immunity against intravaginal SHIV infections by bNAbs delivered via intravenous (i.v.) bolus injection is typically conferred by clearance of the virus from distal tissues.[13,48] This is unsurprising given the significantly higher concentration of bNAb in circulation versus vaginal lumen.[2] Clearly, our local delivery approach of mRNA-expressed bNAbs should be intensively tested in an intravaginal SHIV challenge model in macaques.

Rapid expression kinetics were observed in sheep and rhesus macaque models (less than 4 h) suggesting that aerosolized mRNA transfection may serve as an acute therapeutic intervention at the site of infection. If cervicovaginally expressed bNAbs have the potential to follow the same diffusion and trafficking routes as cell-free virions, mRNA transfection may be used in combination with parenteral bNAbs to prevent or attenuate initial virus seeding. PET/CT tracking of mRNA further suggests that mRNA reaches systemic lymph nodes within 70 min after aerosol administration; mRNA expression in these secondary lymphoid organs was not evaluated in this study, but future work will explore this observation further. The majority of mRNA remained in the FRT, which is useful in preventing liver expression and toxicity, and localized antibody production may mitigate development of anti-antibody responses. While significant cytokine responses to mRNA delivery in the lung were not observed in our previous work, future studies will assess the safety/toxicity profile of local mRNA delivery to the cervicovaginal mucosa.[21]

Another benefit of this platform lies in the relatively simple formulation with water. When lyophilized, mRNA is extremely stable and is not dependent on cold-chain storage conditions.[49] The robust transfections we observed, simplicity of the aerosolizer, and low cost of IVT mRNA, all support the potential for on-demand use as a self-applied prophylaxis technique.

Additionally, this platform provides a means to combine multiple therapeutics into a single formulation, as reported here with the simultaneous expression of antibody HCs and LCs, and in our previous study on respiratory syncytial virus (RSV),[21] overcoming the limits imposed by the use of delivery vehicles.[30] Combinations of neutralizing or non-neutralizing antibodies that target non-overlapping gp120 and gp41 epitopes could be co-expressed, either as single-chain antibodies such as single-domain camelids, ScFv, and ScFvFc or as antibodies that share a HC or LC amino acid sequence.[50,51] There is some evidence that chronic controllers do this naturally and develop multiple bNAbs targeting different, non-overlapping sites on *Env* to suppress virus.[9] Using optimal CD4bs, V3, and MPER targeting antibodies simultaneously could result in $IC_{50}$'s as low as 0.01 µg/mL, with 100% breadth.[52] Alternatively, bispecific and trispecific ScFv antibodies consisting of the binding domains of multiple bNAbs, increase the breadth of coverage and display high binding affinities, and can be expressed from a single mRNA transcript comparable in size to the ones used in this study.[53]

Other STIs that correlate with HIV, such as herpes, hepatitis B, and genital warts (human papillomavirus [HPV]), could be concomitantly targeted with relevant antibodies or anti-microbial peptides.

This study suggests a complementary mRNA-based approach to large dose systemic antibody injection to achieve rapid (less than 4 h) and long-lasting (at least 28 days) neutralizing antibody concentrations within the lower female reproductive tract, providing a firm basis and rationale for an *in vivo* SHIV challenge in NHPs, and a possible platform paradigm shift for the prevention and treatment of STIs.

## MATERIALS AND METHODS

### Animals

Yearling nonpregnant female Katahdin ewes were sourced from a farm in Starkville, MS, and housed in the College of Veterinary Medicine Teaching and Research Pastures 11, 12, and 3 at Mississippi State University. All experiments were reviewed and approved by the Mississippi State University IACUC.

Female rhesus macaques (n = 3) used in this study were housed in the BSL2+ housing of the New Iberia Research Center and maintained in accordance with the regulations of the Guide for the Care and Use of Laboratory Animal and the studies were reviewed and approved by the University of Louisiana IACUC. The macaques were fed monkey chow (Purina) supplemented daily with fresh fruit or vegetables with water provided *ad libitum*. All procedures in macaques were conducted under sedation with Telazol and/or ketamine.

### Cell Lines and Virus Culture

Vero and HEK293 cells were obtained from ATCC and cultured in RPMI1640 media supplemented with L glutamine, 10% fetal bovine serum (FBS), and antibiotics.

SHIV162p3 (simian immunodeficiency virus SIVmac239 backbone with an HIV-1 clade B, R5-tropic envelope) was provided by Dr. Nancy Miller, National Institute of Allergy and Infectious Diseases, National Institutes of Health. SHIV2873Nip (SIVmac239 backbone with an HIV-1 clade C, R5-tropic envelope isolated from a Zambian infant) was obtained from Dr. Ruth Ruprecht, the Texas Biomed AIDS Research Program, Texas Biomedical Research Institute. Both viruses were propagated in rhesus peripheral blood mononuclear cells activated with Concanavalin A and recombinant interleukin-2 (IL-2). Virus stocks were titrated in TZM-bl cells to derive the 50% tissue culture infective dose ($TCID_{50}$). For neutralization assay, both viruses were used at a concentration that resulted in $10^5$ relative light units (RLU) with a repeat titration of viral stocks in parallel to each assay.

### Antibodies

For immunostaining, the primary antibodies used were mouse anti-CD63 (Developmental Studies Hybridoma Bank, Cat. No. H5C6), mouse anti-clathrin LC (Biolegend, Cat. No. MMS-423P), mouse anti-caveolin (Abcam, Cat. No. ab17052), LAMP1 (Developmental Studies Hybridoma Bank, Cat. No. H4A3), mouse anti-EEA1 (BD Biosciences, Cat. No. 610456), and rabbit anti-NanoLuc (Promega). All primary antibodies for immunostaining experiments were used at 1 μg/mL. Secondary antibodies used were donkey anti-mouse Alexa Fluor 546 (Life Technologies) and donkey anti-rabbit Alexa Fluor 488 (Life Technologies). All secondary antibodies for immunostaining experiments were used at 8 μg/mL.

For western blot, primary antibodies used were rabbit anti-NanoLuc (Promega), and mouse anti-GAPDH (GeneTex, Cat. No. GT239), diluted to 0.74 μg/mL and 1 μg/mL, respectively, in Odyssey blocking buffer (LI-COR) with 0.1% Tween-20. The secondary antibodies were a donkey anti-mouse IRDye 680RD (LI-COR) and a donkey anti-rabbit IRDye 800 (LI-COR) and were diluted 1:3,000 in Odyssey blocking buffer with 0.1% Tween-20.

### Synthetic mRNA *In Vitro* Transcription

The anchored and secreted PGT121 sequences were ordered as a DNA gBlock from IDT (Integrated DNA Technologies; sequences found in Table S2) containing a 5′ UTR with Kozak sequence, a 3′ UTR derived from the mouse alpha globin sequence, and extensions to allow for Gibson assembly. The sequences were human codon optimized using the IDT website. The gBlock was cloned into a PCR amplified pMA7 vector (primers B1 and B2, Table S3) through Gibson assembly using NEB Builder with 3× molar excess of insert. All reaction transcripts were 0.8% agarose gel purified prior to assembly reaction. Subsequent plasmids from each colony were Sanger sequenced to ensure desired sequence fidelity.

Plasmids were linearized with NotI-HF (New England BioLabs) overnight at 37°C. Linearized templates were purified by sodium acetate (Thermo Fisher Scientific) precipitation before being rehydrated with nuclease free water. IVT was performed overnight at 37°C using the HiScribe T7 kit (NEB) following the manufacturer's instructions (N1-methyl-pseudouridine modified). RNA product was treated with DNase I (Aldevron) for 30 min to remove template and purified using lithium chloride precipitation (Thermo Fisher Scientific). RNA was heat denatured at 65°C for 10 min before being capped with a Cap-1 structure using guanylyl transferase (Aldevron) and 2′-O-methyl-transferase (Aldevron). Transcripts were then polyadenylated enzymatically (Aldevron). mRNA was then purified by lithium chloride precipitation, treated with alkaline phosphatase (NEB), and purified again. Concentrations were measured using a Nanodrop. mRNA stock concentrations were 3–5 mg/mL. Purified RNA products were analyzed by gel electrophoresis to ensure purity.

### Particle Mediated *In Vitro* Transfections

Vero or HEK293 cells were transfected using either Lipofectamine 2000 (Thermo Fisher Scientific) or Neon electroporation system (Invitrogen), according to the manufacturer's instructions, into a 24-well plate (for imaging) and were transfected with the indicated amount of mRNA per 200,000 cells. For mRNA encoding whole IgG, HC and LC mRNAs were combined in a 4:1 mass ratio for equimolar conditions. 20 h post-transfection, cells were fixed and immunostained with or without permeabilization.

### Immunostaining

Vero or HEK293 cells were fixed with 4% paraformaldehyde (PFA) (Electron Microscopy Sciences) for 10 min at room temperature before permeabilization with 0.2% Triton X-100 (Sigma) for 5 min at room temperature. Then, cells were blocked by incubation with 5% BSA (Calbiochem) for 30 min at 37°C before being incubated with primary antibody for 30 min at 37°C. Cells were then washed with PBS and incubated with secondary antibody for 30 min at 37°C. Multiple antibody labeling was performed simultaneously after checking cross-reactivity. Nuclei were then stained with 4',6-diamidino-2-phenylindole (DAPI) (Life Technologies), and coverslips were mounted onto glass slides with Prolong Gold (Life Technologies).

### Sheep Aerosol Delivery

Sheep were sedated using 0.2 mg/kg of xylazine administered i.v. After 3–5 min, animals were positioned in dorsal recumbency on a flat table. A polyethylene vaginal speculum, with an internal diameter of 15 mm, was coated with sterile lubricant and positioned such that the cervical os was visible. Mucus was cleared away by brief surface cleaning with a cotton-tipped applicator. The MADgic, with dosing syringe, was then inserted into the speculum until the distal nozzle of the aerosolizer was 5 mm from the distal opening of the speculum. Hand pressure was then used to spray the mRNA. If the vagina was also being sprayed in the same animal, the speculum was removed in a caudal direction by 2–4 cm, surface cleaning was performed, and the vagina was sprayed with a freshly loaded dose of mRNA. Average volumes delivered ranged from 300–450 μL, depending on the experiment. Upon completion of the procedure, the sheep was placed in sternal recumbency and sedation was reversed via intramuscular administration with 1.4 mg/kg tolazoline when applicable. Speculums were disinfected in 4% chlorhexidine solution and rinsed with distilled water between animals, to minimize potential cross contamination. All medication doses, mRNA doses, times of administration, and other experimental notes were recorded, as required by IACUC at Mississippi State University.

### Sheep Secretion Collection

Sponges (Beaver-Visitec International) were presoaked with 50 μL of 1× PBS. If multiple sites within the lower FRT were sampled simultaneously, the sponges were connected using monofilament suture. With the animals standing and non-sedated, vaginal secretions were collected by positioning the sponge in the desired location using forceps and waiting for 3 min. The sponges were then removed and placed into 1.5 mL tubes and frozen at −80°C until analysis. To extract secretions from the sponges, we first placed the sponges into pre-weighed QiaShredder filters and centrifuged them at 20,000× *g* for 10 min at 4°C. 200 μL of extraction solution (IGEPAL, protease inhibitor in PBS) was added to the sponge and placed on ice for 15 min. The sponge was then spun at 20,000× *g* for a further 30 min. Secretions were aliquoted and stored at −80°C for further analysis.

### IVIS Imaging and Image Analysis

Sheep were euthanized by i.v. barbiturate injection and the female reproductive tract was removed and excised. The tract was cut along a single edge so the entire surface area of the vagina and cervix lay flat. 2 mL of a fresh 1:40 solution of NanoGlo Substrate in 1× PBS was added to the entire vagina and uterus. After 1 min at room temperature, the organ was imaged with an IVIS (IVIS lumina XRMS, Series III; supported through USDA-ARS Biophotonics Initiative #58-6402-3-018). The positive regions were identified from the images and carefully excised. The tissues were then either weighed and snap frozen in liquid nitrogen for protein extraction or placed in 4% paraformaldehyde overnight at 4°C for microscopic analysis.

Acquired luminescence FRT images were analyzed using Living Image Software (PerkinElmer). Relevant regions of interest (ROIs) were identified from background tissue by high-pass thresholding at 10%. The average radiance for each ROI was measured and control average radiance from a negative control sample was used for background subtraction purposes.

### Sheep Tissue Lysate Preparation

Frozen sheep FRT tissues were crushed using a BioPulverizer (BioSpec Products). Radioimmunoprecipitation assay (RIPA) buffer (Thermo Fisher Scientific) was then added at a ratio of 2 μL to 1 mg of tissue. This solution was then further homogenized using a bead mill (Next Advance) before being centrifuged at 16,000× *g* for 20 min. Lysate supernatants were then aliquoted and stored frozen at −80°C. Lysate protein concentrations were determined using a bicinchoninic acid assay (BCA, Pierce).

### *Macaque Aerosol Delivery*

Female rhesus macaques (n = 3) were used in this study. Rhesus macaques were sedated with Telazol/ketamine and placed in ventral recumbency. PGT121 mRNA solution, in molecular grade nuclease-free $H_2O$, was loaded into a 1 mL high-pressure syringe (Medline). The syringe was attached to the MADgic Laryngo-Tracheal Mucosal Atomization Device (Teleflex). A modified 3 mL syringe was used as a speculum to visualize the cervix. Mucus from the cervicovaginal lumen was cleared away by brief surface cleaning with a cotton-tipped applicator. The MADgic, with dosing syringe, was then inserted into the tube until the distal nozzle of the aerosolizer was 5 mm from the distal opening of the speculum. Hand pressure was then used to spray the mRNA solution. If the vagina was also being sprayed in the same macaque, the speculum was removed in a caudal direction by 2–4 cm, and the vagina was sprayed with a freshly loaded dose of mRNA. Each 250 μg dose was 300 μL, while the 400 and 1,000 μg dose was 150 μL each to minimize leakage of excess solution.

### *mRNA Radiolabeling and Distribution by PET/CT Imaging*

To study the bio-distribution of IVT mRNA via whole-body PET-CT, we annealed radionuclide-labeled antisense oligonucleotides to the mRNA before delivery, as previously described.[31,32,39] Briefly, two 2′*O*-methyl RNA/DNA chimeric oligos (Biosearch Technologies)

complementary to the mRNA 3′ UTR were purchased with the following sequences: MT1 5′-Thiol-XTTTTTX**GCAAGCCCCGCA GAAG**X-3′ and MT2 5′-Thiol-TXTTATTX**AGAGAAGAAGGG CA**X**GG-3′** where the boldface indicates 2′-*O*-methyl RNA and X indicates T(C6-Amino) modifications.

For chelator conjugation, the 5′ disulfide was reduced by incubation with tris(2-carboxyethyl)phosphine(TCEP) (5 mM) (Thermo Fisher Scientific). Oligonucleotides were then repeatedly diluted in 0.1 M chelexed phosphate buffer pH 7.3 and filtered (3 kDa MWCO, Millipore) to remove the reducing agent. Oligonucleotides were then modified by incubation with 1,4,7,10-tetraazacyclododecane-1,4,7,10-tetraacetic acid (DOTA)-maleimide and DOTA-NHS ester (10× and 50× molar excess, respectively, Macrocyclics) for 6 h at room temperature under gentle agitation.

Unbound chelators were removed by centrifugal filtration (3 kDa MWCO) in 0.1 M chelexed-phosphate buffer, and the individual oligos were quantified by Nanodrop, aliquoted, and lyophilized. Oligos were then resuspended in 0.1 M chelexed ammonium acetate pH 5.5 and incubated with $^{64}$Cu for 1 h at 37°C. Unbound $^{64}$Cu was removed by centrifugal filtration (3kDA MWCO) in 0.1 M chelexed-phosphate buffer.

To pre-label IVT mRNA before delivery, we annealed mRNA to 0.7× molar excess probes in a thermal cycler with the following optimized protocol: 80°C for 2 min, gradients from 80°C to 25°C in 30 s steps (1 deg/step), and 25°C for 2 min. The mRNA was immediately resuspended in saline and delivered.

The aerosolized dose of radiolabeled mRNA delivered to rhesus macaques was 0.2 mCi per macaque, delivered in two sequential 300 μL doses using the Teleflex atomizer. PET/CT scanning was undertaken at 70 min, 4 h, 24 h, and 48 h post administration using a Philips Gemini TF64 clinical PET/CT scanner.

All quantitative software analysis was completed from DICOM formatted images using MIM Software (Cleveland, OH). A high-pass thresholding filter set at 28% (i.e., only the highest 72% of signal within a given region) was used to assign ROIs for each lymph node. These volumetric regions of interest were used to report the total SUV. To account for instrument parameters that might have altered between imaging sessions, we used contralateral muscle SUV average values for a circumscribed ROI to normalize readings. The video clip in Video S1 displaying a 3D reconstruction of the PET/CT data was accomplished through Amira software (Thermo Fisher Scientific, CA).

### SHIV Neutralization Titers

The neutralization activity of antibody in macaque vaginal secretions against either SHIV162p3 (clade B) or SHIV2873Nip (clade C) was measured using the reference protocol of the luciferase-based HIV-1 neutralization assay in TZM-bl cells (Dr. Montefiori laboratory, Duke University). Briefly, 50 μL of 5-fold serial dilutions of vaginal secretions and 50 μL of titrated virus ($10^5$ RLU) were incubated for 1 h at 37°C in a 96-well flat-bottom plate. Next, 100 μL of TZM-bl cells (1 × $10^4$/well) in 10% DMEM growth medium containing 30 μg/mL diethylaminoethyl (DEAE) dextran (Sigma-Aldrich) were added to each well, and the 96-well plates were incubated for 48 h. Assay controls included TZM-bl cells alone (cell control, no virus) and TZM-bl cells with virus only (virus control, no test reagent). At 48 h, the cells were lysed and luciferase activity was measured using a BioTek Synergy HT multimode microplate reader. The average background luminescence (RLU) from cell control wells was subtracted from the luminescence for each experimental well, and infectivity curves were generated using GraphPad Prism (v7.01) software, where values from the experimental wells were compared against the value from virus control wells. The 50% inhibitory concentration ($IC_{50}$) was calculated based on the vaginal secretions dilution that caused a 50% reduction of RLU compared to the virus control wells after subtraction of cell control RLU.

To measure the neutralization activity of mRNA-expressed antibodies, we seeded Vero cells into 75 $cm^2$ culture flasks and transfected with either aPGT121 or sPGT121 HC mRNA and NanoLuc or non-NanoLuc PGT121 LC mRNA. For aPGT121 constructs, the cells were lysed using RIPA buffer and clarified by centrifugation for 20 min at 18,000× *g* at 4°C. The supernatant was then collected and stored at −80°C until further use. For sPGT121 constructs, the culture media supernatant was concentrated using a 10 kDa molecular weight cutoff (MWCO) centrifugal filter before being stored at −80°C until further use. The cell lysates (for aPGT121) and cell supernatants (for sPGT121) were purified using a NAb Protein A Plus spin column (Pierce) before being assayed for neutralization activity as described above. mRNA-expressed antibodies were compared to PGT121-N (a gift from Mapp Biopharmaceutical). Neutralization activity from all constructs was compared using molarity to account for molecular weight difference due to the inclusion of the membrane anchor or NanoLuc reporter.

### SHIV ADCC Assay

ADCC was assessed as previously described.[54] Briefly, CEM.NKR.CCR5.CD4$^+$-Luc target cells were infected with 50 ng SHIV162p3 or SHIV2873Nip by spinoculation and cultured for 4 days. 2-fold serial dilutions of each PGT antibody, either mRNA-expressed (aPGT121-NLuc, aPGT121, sPGT121-NLuc, or sPGT121) were added to the infected targets for 20 min at room temperature. An NK cell line CD16-KHYG-1, as effector cells, were added at a 10:1 effector to target ratio and these were incubated for additional 8 h. The cells were then lysed and luciferase activity (RLU) was measured using a luminometer (Synergy HT, Bio-Tek).

### Macaque Explant SHIV Challenge

At 24 h post transfection, biopsies from the indicated regions of the FRT in transfected macaques were collected. Biopsies were cut to similar size, between 2.5 and 3 mm in diameter. Replicate biopsies at a given dose represent separate biopsies from an area, rather than single biopsies dissected into multiple pieces. Tissues were

then washed 3× with 1× PBS, incubated with 5.5 × $10^4$ TCID50 of SHIV162p3 for 2 h at 37°C, washed a further 3×, and then cultured on collagen sponges (Surgifoam) in 10% FBS supplemented DMEM media. Supernatant from the cultures was collected at the indicated time points and frozen, until p27 ELISA results could be attained by ELISA.

### NanoLuc Luciferase gp120 ELISA

The gp120 ELISA protocol was adapted from Parren et al.[18] Recombinant $gp120_{JR\text{-}FL}$ (MyBioSource) was coated to the wells of a microtiter plate (Corning) at a concentration of 2 μg/mL by incubation overnight at 4°C in 1× PBS. The plates were washed four times with PBS–0.05% Tween-20 and blocked with 3% BSA. Following washing, vaginal secretions or purified PGT121-NanoLuc stocks were applied to the plate and incubated for 2 h at 37°C. A PGT121-NanoLuc antibody standard curve was run on each plate, as necessary. After washing, a 1:50 ratio of NanoGlo in 1× PBS was added to each well. After 1 min, a BioTek Synergy H4 Microplate reader set at an emission spectrum of 450 nm and 2 s integration time was used to record the luminescence in each sample.

### *Western Blot*

The indicated quantity of tissue lysates or secretions were mixed with 4× SDS loading buffer (LI-COR Biosciences), boiled for 10 min at 70°C, chilled on ice, and loaded into wells of a Bolt 4%–12% Bis-Tris Bolt precast gel (Life Technologies) alongside a molecular weight marker (LI-COR). Gel was run in a Mini Gel Tank system (Life Technologies) in 1X 3-morpholinopropane-1-sulfonic acid (MOPS) running buffer (Life Technologies) at a constant 200 V for 32 min. Protein was then transferred to 0.45 μm pore nitrocellulose membranes (Life Technologies) in 1× Bolt western transfer buffer (Life Technologies) at a constant 12 V for 1 h using a Mini gel blot module (Life Technologies).

Nonspecific binding to blot was blocked using PBS Odyssey blocking buffer (LI-COR) at room temperature. Primary antibody was then applied in blocking solution with 0.1% Tween-20 (VWR) and allowed to incubate overnight at 4°C. Blots were washed three times with 1× PBS containing 0.1% Tween-20 (PBST). Secondary antibody was then applied and allowed to incubate for 1 h before blots were again washed three times with PBST. Blots were imaged using an Odyssey CLx IR scanner (LI-COR). Only linear contrast enhancements were performed for the final representative images.

For quantification, a standard curve dilution series of purified PGT121 was loaded in the same gel as the samples in all cases. A linear regression with interpolation was then performed on the densitometry of the detected band and samples.

### Statistical Analysis

Results were plotted, and statistical analyses were performed using Prism 8 (GraphPad, La Jolla, CA). Power analysis was performed to ensure adequate sample size for experiments. Hypothesis tests were chosen and performed as appropriate, indicated in the figure captions.

### Data Availability

The authors declare that all other data supporting the findings of this study are available within the paper and its Supplemental Information files. PET/CT scanner acquisition settings can be attained by writing to the corresponding author, P.J.S.

## SUPPLEMENTAL INFORMATION

Supplemental Information can be found online at https://doi.org/10.1016/j.ymthe.2020.01.002.

## AUTHOR CONTRIBUTIONS

K.E.L., D.V., F.V., A.R.W., and P.J.S. contributed to the design of the study. K.E.L., D.V., H.E.P., and P.M.T. contributed to synthetic mRNA design and synthesis. K.E.L., C.Z., and D.V. contributed to *in vitro* cell-based experiments. H.K., M.T., P.B., A.R.W., K.E.L., D.V., and A.K.O. contributed to the sheep *in vivo* transfection experiments. P.B., S.B.P., K.E.L., M.T., H.K., D.V., and J.M.F. contributed to sheep *ex vivo* tissue imaging. K.E.L., M.A., D.V., and F.V. contributed to rhesus macaque *in vivo* transfection experiments. M.A., F.V., K.E.L., and C.Z. contributed to PET/CT rhesus macaque imaging experiments. D.V. and K.E.L. contributed to secretion and neutralization antibody quantification. P.X., K.E.L., M.A., E.L.B., and F.V. contributed to SHIV challenge and neutralization studies. K.E.L., D.V., C.Z., and P.J.S. drafted and edited the manuscript. All authors approved the final manuscript.

## CONFLICTS OF INTEREST

The authors declare no competing interests.

## ACKNOWLEDGMENTS

The IVIS Lumina use at Mississippi State University was generously supported by the USDA-ARS Biophotonics Initiative #58-6402-3-018. The authors would like to acknowledge Leslie Scruggs of Scrugg's Dorpers farm for providing the sheep. The authors are also indebted to the veterinary and research personnel at Mississippi State University and the New Iberia Research Center for their expert help in performing the animal experiments. The Petit Institute Core at Georgia Tech, particularly Andrew Shaw, provided guidance and resources for confocal microscopy.

## REFERENCES

1. UNAIDS. (2019). Global HIV & AIDS statistics — 2019 fact sheet. https://www.unaids.org/en/resources/fact-sheet.
2. Brandenberg, O.F., Magnus, C., Rusert, P., Günthard, H.F., Regoes, R.R., and Trkola, A. (2017). Predicting HIV-1 transmission and antibody neutralization efficacy in vivo from stoichiometric parameters. PLoS Pathog. *13*, e1006313.
3. Miller, W.C., Rosenberg, N.E., Rutstein, S.E., and Powers, K.A. (2010). Role of acute and early HIV infection in the sexual transmission of HIV. Curr. Opin. HIV AIDS *5*, 277–282.
4. Zimmerman, P.A., Buckler-White, A., Alkhatib, G., Spalding, T., Kubofcik, J., Combadiere, C., Weissman, D., Cohen, O., Robbert, A., Lam, G., et al. Inherited Resistance to HIV-1 Conferred by an Inactivating Mutation in CC Chemokine

Receptor 5: Studies in Populations with Contrasting Clinical Phenotypes, Defined Racial Background, and Quantified Risk. Molecular Medicine 3, 23–36.

5. Stieh, D.J., Maric, D., Kelley, Z.L., Anderson, M.R., Hattaway, H.Z., Beilfuss, B.A., Rothwangl, K.B., Veazey, R.S., and Hope, T.J. (2014). Vaginal challenge with an SIV-based dual reporter system reveals that infection can occur throughout the upper and lower female reproductive tract. PLoS Pathog. *10*, e1004440.

6. Anderson, D.J. (2010). Finally, a macaque model for cell-associated SIV/HIV vaginal transmission. J. Infect. Dis. *202*, 333–336.

7. Barouch, D.H., Ghneim, K., Bosche, W.J., Li, Y., Berkemeier, B., Hull, M., Bhattacharyya, S., Cameron, M., Liu, J., Smith, K., et al. (2016). Rapid Inflammasome Activation following Mucosal SIV Infection of Rhesus Monkeys. Cell *165*, 656–667.

8. Astronomo, R.D., Santra, S., Ballweber-Fleming, L., Westerberg, K.G., Mach, L., Hensley-McBain, T., Sutherland, L., Mildenberg, B., Morton, G., Yates, N.L., et al. (2016). Neutralization Takes Precedence Over IgG or IgA Isotype-related Functions in Mucosal HIV-1 Antibody-mediated Protection. EBioMedicine *14*, 97–111.

9. Freund, N.T., Wang, H., Scharf, L., Nogueira, L., Horwitz, J.A., Bar-On, Y., Golijanin, J., Sievers, S.A., Sok, D., Cai, H., et al. (2017). Coexistence of potent HIV-1 broadly neutralizing antibodies and antibody-sensitive viruses in a viremic controller. Sci. Transl. Med. *9*, eaal2144.

10. Julg, B., Sok, D., Schmidt, S.D., Abbink, P., Newman, R.M., Broge, T., Linde, C., Nkolola, J., Le, K., Su, D., et al. (2017). Protective Efficacy of Broadly Neutralizing Antibodies with Incomplete Neutralization Activity against Simian-Human Immunodeficiency Virus in Rhesus Monkeys. J. Virol. *91*, e01187–e17.

11. Julg, B., Liu, P.-T., Wagh, K., Fischer, W.M., Abbink, P., Mercado, N.B., Whitney, J.B., Nkolola, J.P., McMahan, K., Tartaglia, L.J., et al. (2017). Protection against a mixed SHIV challenge by a broadly neutralizing antibody cocktail. Sci. Transl. Med. *9*, eaao4235.

12. Hessell, A.J., Rakasz, E.G., Poignard, P., Hangartner, L., Landucci, G., Forthal, D.N., Koff, W.C., Watkins, D.I., and Burton, D.R. (2009). Broadly neutralizing human anti-HIV antibody 2G12 is effective in protection against mucosal SHIV challenge even at low serum neutralizing titers. PLoS Pathog. *5*, e1000433.

13. Hessell, A.J., Jaworski, J.P., Epson, E., Matsuda, K., Pandey, S., Kahl, C., Reed, J., Sutton, W.F., Hammond, K.B., Cheever, T.A., et al. (2016). Early short-term treatment with neutralizing human monoclonal antibodies halts SHIV infection in infant macaques. Nat. Med. *22*, 362–368.

14. Caskey, M., Klein, F., Lorenzi, J.C.C., Seaman, M.S., West, A.P., Jr., Buckley, N., Kremer, G., Nogueira, L., Braunschweig, M., Scheid, J.F., et al. (2015). Viraemia suppressed in HIV-1-infected humans by broadly neutralizing antibody 3BNC117. Nature *522*, 487–491.

15. Caskey, M., Schoofs, T., Gruell, H., Settler, A., Karagounis, T., Kreider, E.F., Murrell, B., Pfeifer, N., Nogueira, L., Oliveira, T.Y., et al. (2017). Antibody 10-1074 suppresses viremia in HIV-1-infected individuals. Nat. Med. *23*, 185–191.

16. Scheid, J.F., Horwitz, J.A., Bar-On, Y., Kreider, E.F., Lu, C.-L., Lorenzi, J.C.C., Feldmann, A., Braunschweig, M., Nogueira, L., Oliveira, T., et al. (2016). HIV-1 antibody 3BNC117 suppresses viral rebound in humans during treatment interruption. Nature *535*, 556–560.

17. Nishimura, Y., Gautam, R., Chun, T.-W., Sadjadpour, R., Foulds, K.E., Shingai, M., Klein, F., Gazumyan, A., Golijanin, J., Donaldson, M., et al. (2017). Early antibody therapy can induce long-lasting immunity to SHIV. Nature *543*, 559–563.

18. Parren, P.W.H.I., Marx, P.A., Hessell, A.J., Luckay, A., Harouse, J., Cheng-Mayer, C., Moore, J.P., and Burton, D.R. (2001). Antibody protects macaques against vaginal challenge with a pathogenic R5 simian/human immunodeficiency virus at serum levels giving complete neutralization in vitro. J. Virol. *75*, 8340–8347.

19. Veazey, R.S., Shattock, R.J., Pope, M., Kirijan, J.C., Jones, J., Hu, Q., Ketas, T., Marx, P.A., Klasse, P.J., Burton, D.R., and Moore, J.P. (2003). Prevention of virus transmission to macaque monkeys by a vaginally applied monoclonal antibody to HIV-1 gp120. Nat. Med. *9*, 343–346.

20. Sherwood, J.K., Zeitlin, L., Chen, X., Whaley, K.J., Cone, R.A., and Saltzman, W.M. (1996). Residence half-life of IgG administered topically to the mouse vagina. Biol. Reprod. *54*, 264–269.

21. Tiwari, P.M., Vanover, D., Lindsay, K.E., Bawage, S.S., Kirschman, J.L., Bhosle, S., Lifland, A.W., Zurla, C., and Santangelo, P.J. (2018). Engineered mRNA-expressed antibodies prevent respiratory syncytial virus infection. Nat. Commun. *9*, 3999.

22. Barry, J.S., and Anthony, R.V. (2008). The pregnant sheep as a model for human pregnancy. Theriogenology *69*, 55–67.

23. Cissik, J.H., Ehler, W.J., Hankins, G.D., and Snyder, R.R. (1991). Cardiopulmonary reference standards in the pregnant sheep (Ovis aries): a comparative study of ovine and human physiology in obstetrics. Comp. Biochem. Physiol. A Comp. Physiol. *100*, 877–880.

24. Holt, J.D.S., Cameron, D., Dias, N., Holding, J., Muntendam, A., Oostebring, F., Dreier, P., Rohan, L., and Nuttall, J. (2015). The sheep as a model of preclinical safety and pharmacokinetic evaluations of candidate microbicides. Antimicrob. Agents Chemother. *59*, 3761–3770.

25. Vincent, K.L., Bourne, N., Bell, B.A., Vargas, G., Tan, A., Cowan, D., Stanberry, L.R., Rosenthal, S.L., and Motamedi, M. (2009). High resolution imaging of epithelial injury in the sheep cervicovaginal tract: a promising model for testing safety of candidate microbicides. Sex. Transm. Dis. *36*, 312–318.

26. Xiao, P., Gumber, S., Marzinke, M.A., Date, A.A., Hoang, T., Hanes, J., Ensign, L.M., Wang, L., Rohan, L., Fuchs, E.J., et al. (2017). Hypo-osmolar Formulation of Tenofovir (TFV) Enema Promotes Uptake and Metabolism of TFV in Tissues, Leading to Prevention of SHIV/SIV Infection. Antimicrob. Agents Chemother. *62*, e01644–17.

27. Deering, R.P., Kommareddy, S., Ulmer, J.B., Brito, L.A., and Geall, A.J. (2014). Nucleic acid vaccines: prospects for non-viral delivery of mRNA vaccines. Expert Opin. Drug Deliv. *11*, 885–899.

28. Pollard, C., Rejman, J., De Haes, W., Verrier, B., Van Gulck, E., Naessens, T., De Smedt, S., Bogaert, P., Grooten, J., Vanham, G., and De Koker, S. (2013). Type I IFN counteracts the induction of antigen-specific immune responses by lipid-based delivery of mRNA vaccines. Mol. Ther. *21*, 251–259.

29. Ensign, L.M., Hoen, T.E., Maisel, K., Cone, R.A., and Hanes, J.S. (2013). Enhanced vaginal drug delivery through the use of hypotonic formulations that induce fluid uptake. Biomaterials *34*, 6922–6929.

30. Ensign, L.M., Cone, R., and Hanes, J. (2014). Nanoparticle-based drug delivery to the vagina: a review. J. Control. Release *190*, 500–514.

31. Kirschman, J.L., Bhosle, S., Vanover, D., Blanchard, E.L., Loomis, K.H., Zurla, C., Murray, K., Lam, B.C., and Santangelo, P.J. (2017). Characterizing exogenous mRNA delivery, trafficking, cytoplasmic release and RNA-protein correlations at the level of single cells. Nucleic Acids Res. *45*, e113–e113.

32. Loomis, K.H., Lindsay, K.E., Zurla, C., Bhosle, S.M., Vanover, D.A., Blanchard, E.L., et al. (2018). In Vitro Transcribed mRNA Vaccines with Programmable Stimulation of Innate Immunity. Bioconjug. Chem. *29*, 3072–3083.

33. Bhosle, S.M., Loomis, K.H., Kirschman, J.L., Blanchard, E.L., Vanover, D.A., Zurla, C., Habrant, D., Edwards, D., Baumhof, P., Pitard, B., and Santangelo, P.J. (2018). Unifying in vitro and in vivo IVT mRNA expression discrepancies in skeletal muscle via mechanotransduction. Biomaterials *159*, 189–203.

34. Du, Y., Pattnaik, A.K., Song, C., Yoo, D., and Li, G. (2012). Glycosyl-phosphatidylinositol (GPI)-anchored membrane association of the porcine reproductive and respiratory syndrome virus GP4 glycoprotein and its co-localization with CD163 in lipid rafts. Virology *424*, 18–32.

35. Nickells, M.W., Alvarez, J.I., Lublin, D.M., and Atkinson, J.P. (1994). Characterization of DAF-2, a high molecular weight form of decay-accelerating factor (DAF; CD55), as a covalently cross-linked dimer of DAF-1. J. Immunol. *152*, 676–685.

36. von Bredow, B., Arias, J.F., Heyer, L.N., Moldt, B., Le, K., Robinson, J.E., Zolla-Pazner, S., Burton, D.R., and Evans, D.T. (2016). Comparison of Antibody-Dependent Cell-Mediated Cytotoxicity and Virus Neutralization by HIV-1 Env-Specific Monoclonal Antibodies. J. Virol. *90*, 6127–6139.

37. Janes, K.A. (2015). An analysis of critical factors for quantitative immunoblotting. Sci. Signal. *8*, rs2.

38. Brodbeck, W.G., Mold, C., Atkinson, J.P., and Medof, M.E. (2000). Cooperation between decay-accelerating factor and membrane cofactor protein in protecting cells from autologous complement attack. J. Immunol. *165*, 3999–4006.

39. Lindsay, K.E., Bhosle, S.M., Zurla, C., Beyersdorf, J., Rogers, K.A., Vanover, D., et al. Visualization of early events in mRNA vaccine delivery in non-human primates via PET-CT and near-infrared imaging.. Nat. Biomed. Eng. 3, 371–380.

40. Siddappa, N.B., Song, R., Kramer, V.G., Chenine, A.-L., Velu, V., Ong, H., Rasmussen, R.A., Grisson, R.D., Wood, C., Zhang, H., et al. (2009). Neutralization-sensitive R5-tropic simian-human immunodeficiency virus SHIV-2873Nip, which carries env isolated from an infant with a recent HIV clade C infection. J. Virol. *83*, 1422–1432.

41. Feige, M.J., Groscurth, S., Marcinowski, M., Shimizu, Y., Kessler, H., Hendershot, L.M., and Buchner, J. (2009). An unfolded CH1 domain controls the assembly and secretion of IgG antibodies. Mol. Cell *34*, 569–579.

42. Acartürk, F. (2009). Mucoadhesive vaginal drug delivery systems. Recent Pat. Drug Deliv. Formul. *3*, 193–205.

43. Garg, A.B., Nuttall, J., and Romano, J. (2009). The future of HIV microbicides: challenges and opportunities. Antivir. Chem. Chemother. *19*, 143–150.

44. McKinnon, L.R., Liebenberg, L.J., Yende-Zuma, N., Archary, D., Ngcapu, S., Sivro, A., Nagelkerke, N., Garcia Lerma, J.G., Kashuba, A.D., Masson, L., et al. (2018). Genital inflammation undermines the effectiveness of tenofovir gel in preventing HIV acquisition in women. Nat. Med. *24*, 491–496.

45. Sharom, F.J., and Radeva, G. (2004). GPI-anchored protein cleavage in the regulation of transmembrane signals. Subcell Biochem. *37*, 285–315.

46. Speare, J.O., Offerdahl, D.K., Hasenkrug, A., Carmody, A.B., and Baron, G.S. (2010). GPI anchoring facilitates propagation and spread of misfolded Sup35 aggregates in mammalian cells. EMBO J. *29*, 782–794.

47. Kooyman, D.L., Byrne, G.W., McClellan, S., Nielsen, D., Tone, M., Waldmann, H., Coffman, T.M., McCurry, K.R., Platt, J.L., and Logan, J.S. (1995). In vivo transfer of GPI-linked complement restriction factors from erythrocytes to the endothelium. Science *269*, 89–92.

48. Liu, J., Ghneim, K., Sok, D., Bosche, W.J., Li, Y., Chipriano, E., Berkemeier, B., Oswald, K., Borducchi, E., Cabral, C., et al. (2016). Antibody-mediated protection against SHIV challenge includes systemic clearance of distal virus. Science *353*, 1045–1049.

49. Bonnet, J., Colotte, M., Coudy, D., Couallier, V., Portier, J., Morin, B., and Tuffet, S. (2010). Chain and conformation stability of solid-state DNA: implications for room temperature storage. Nucleic Acids Res. *38*, 1531–1546.

50. Kong, R., Louder, M.K., Wagh, K., Bailer, R.T., deCamp, A., Greene, K., Gao, H., Taft, J.D., Gazumyan, A., Liu, C., et al. (2015). Improving neutralization potency and breadth by combining broadly reactive HIV-1 antibodies targeting major neutralization epitopes. J. Virol. *89*, 2659–2671.

51. Doria-Rose, N.A., Louder, M.K., Yang, Z., O'Dell, S., Nason, M., Schmidt, S.D., McKee, K., Seaman, M.S., Bailer, R.T., and Mascola, J.R. (2012). HIV-1 neutralization coverage is improved by combining monoclonal antibodies that target independent epitopes. J. Virol. *86*, 3393–3397.

52. Bouvin-Pley, M., Morgand, M., Meyer, L., Goujard, C., Moreau, A., Mouquet, H., Nussenzweig, M., Pace, C., Ho, D., Bjorkman, P.J., et al. (2014). Drift of the HIV-1 envelope glycoprotein gp120 toward increased neutralization resistance over the course of the epidemic: a comprehensive study using the most potent and broadly neutralizing monoclonal antibodies. J. Virol. *88*, 13910–13917.

53. Steinhardt, J.J., Guenaga, J., Turner, H.L., McKee, K., Louder, M.K., O'Dell, S., Chiang, C.I., Lei, L., Galkin, A., Andrianov, A.K., et al. (2018). Rational design of a trispecific antibody targeting the HIV-1 Env with elevated anti-viral activity. Nat. Commun. *9*, 877.

54. Alpert, M.D., Heyer, L.N., Williams, D.E.J., Harvey, J.D., Greenough, T., Allhorn, M., and Evans, D.T. (2012). A novel assay for antibody-dependent cell-mediated cytotoxicity against HIV-1- or SIV-infected cells reveals incomplete overlap with antibodies measured by neutralization and binding assays. J. Virol. *86*, 12039–12052.

YMTHE, Volume *28*

## Supplemental Information

# Aerosol Delivery of Synthetic mRNA to Vaginal Mucosa Leads to Durable Expression of Broadly Neutralizing Antibodies against HIV

**Kevin E. Lindsay, Daryll Vanover, Merrilee Thoresen, Heath King, Peng Xiao, Peres Badial, Mariluz Araínga, Seong Bin Park, Pooja M. Tiwari, Hannah E. Peck, Emmeline L. Blanchard, Jean M. Feugang, Alicia K. Olivier, Chiara Zurla, Francois Villinger, Amelia R. Woolums, and Philip J. Santangelo**

**Supplementary Materials:**

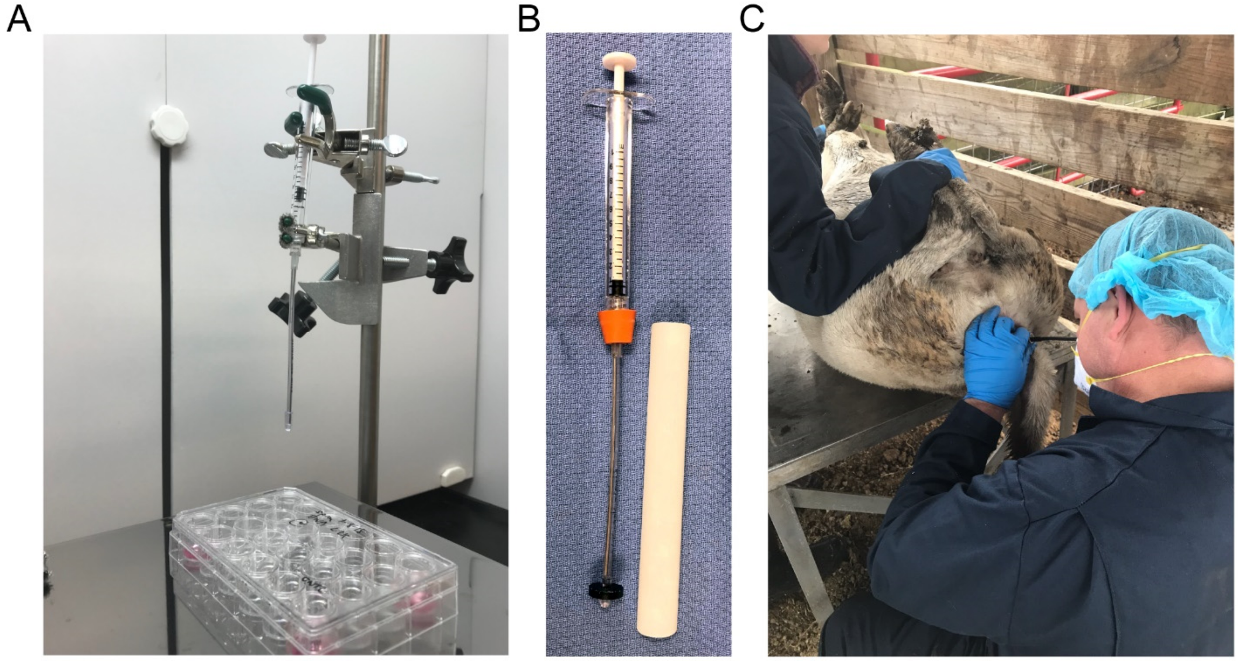

**Fig. S1. *In vitro* and *in vivo* aerosolization apparatus and field application example. (A)** *In vitro* aerosol apparatus. Height was adjusted using a scissor-lift lab jack. Teleflex was held using clamps and actuated by hand. **(B)** *In vivo* Teleflex apparatus with speculum. Teleflex syringe was modified using flat and conical rubber washer to allow for consistent centering and distance when placed in the speculum. The speculum was a cut and sanded piece of plastic tubing. **(C)** Treatment of sheep in field. Sheep were sedated and placed in dorsal recumbency to allow for easy mRNA application.

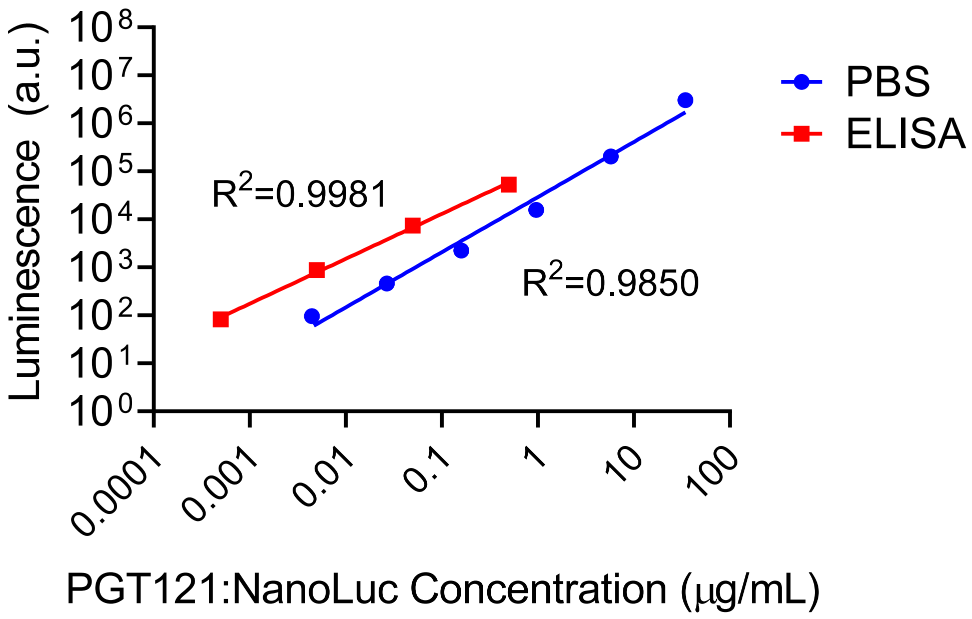


**Fig. S2. HIV gp120 ELISA using PGT121:NanoLuc Luminescence**. Luminescence of a range of concentrations of purified PGT121:NanoLuc were either measured in solution (PBS) or after capture via gp120 coated plate (ELISA). Linear regression was performed on log-log transformed data.

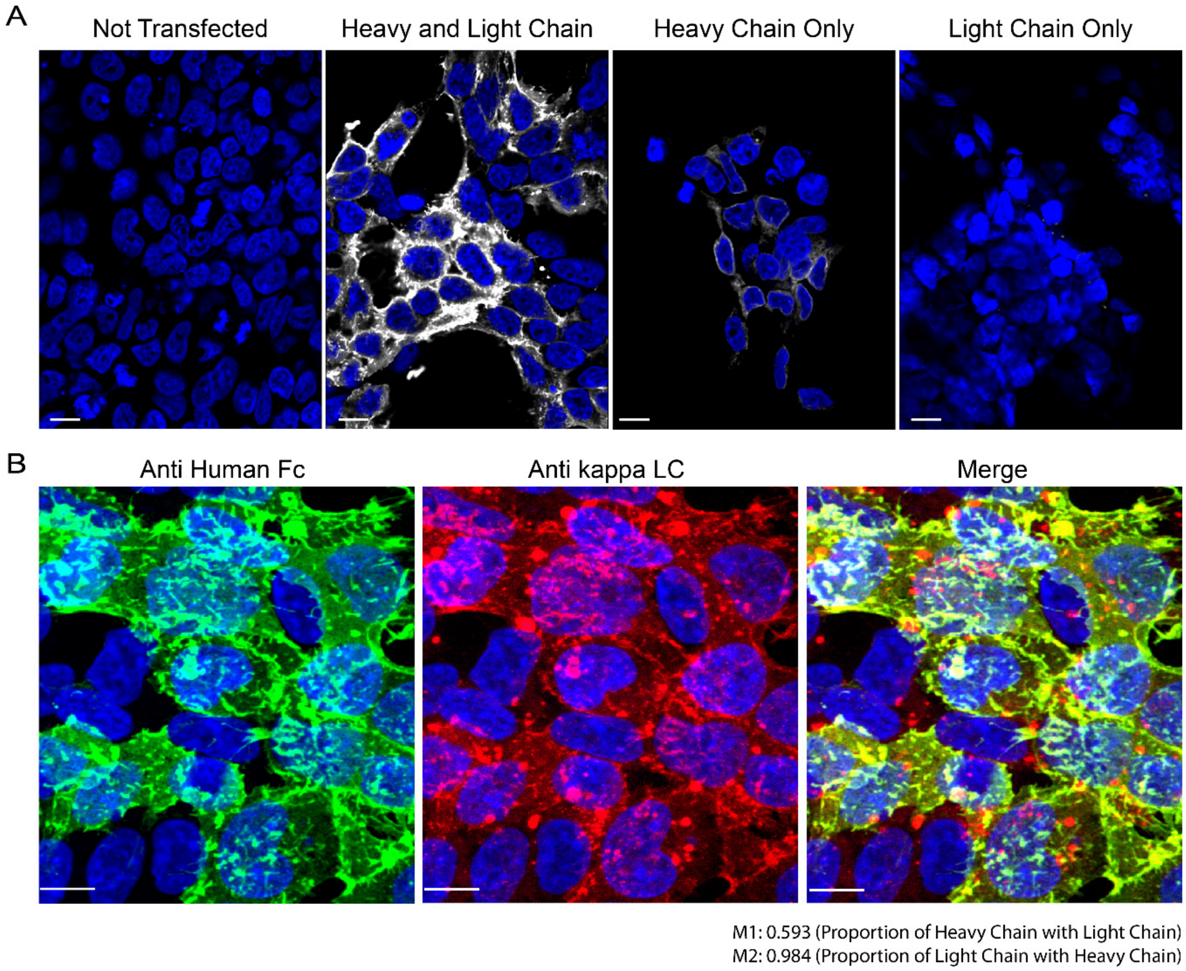


**Fig. S3. PGT121 LC and HC localization to cell surface after mRNA transfection. (A)** HEK293 cells were transfected using 1ug of total synthetic PGT121 mRNA, in the following conditions: 1) as a 4:1 ratio of HC to LC encoding transcripts; 2) HC encoding transcripts only; or 3) LC encoding transcripts only. 24 hours post-transfection, the cells were fixed, not permeabilized, and immunostained with anti-human antibody (Jackson). White - anti-human antibody (Jackson), Blue -DAPI. Scale bars are 10µm. **(B)** HEK293 cells were transfected with 1ug of HC and LC PGT121 mRNA, at a 4:1 ratio. After 24 hours, the cells were fixed and immunostained with anti-human Fc and anti-kappa light chain antibodies. Colocalization coefficients indicate that 98% of the LC protein overlaps with HC protein. Green – anti-human antibody (Jackson), Red – anti-kappa light chain (BD), Blue – DAPI. Scale bars are 10µm.

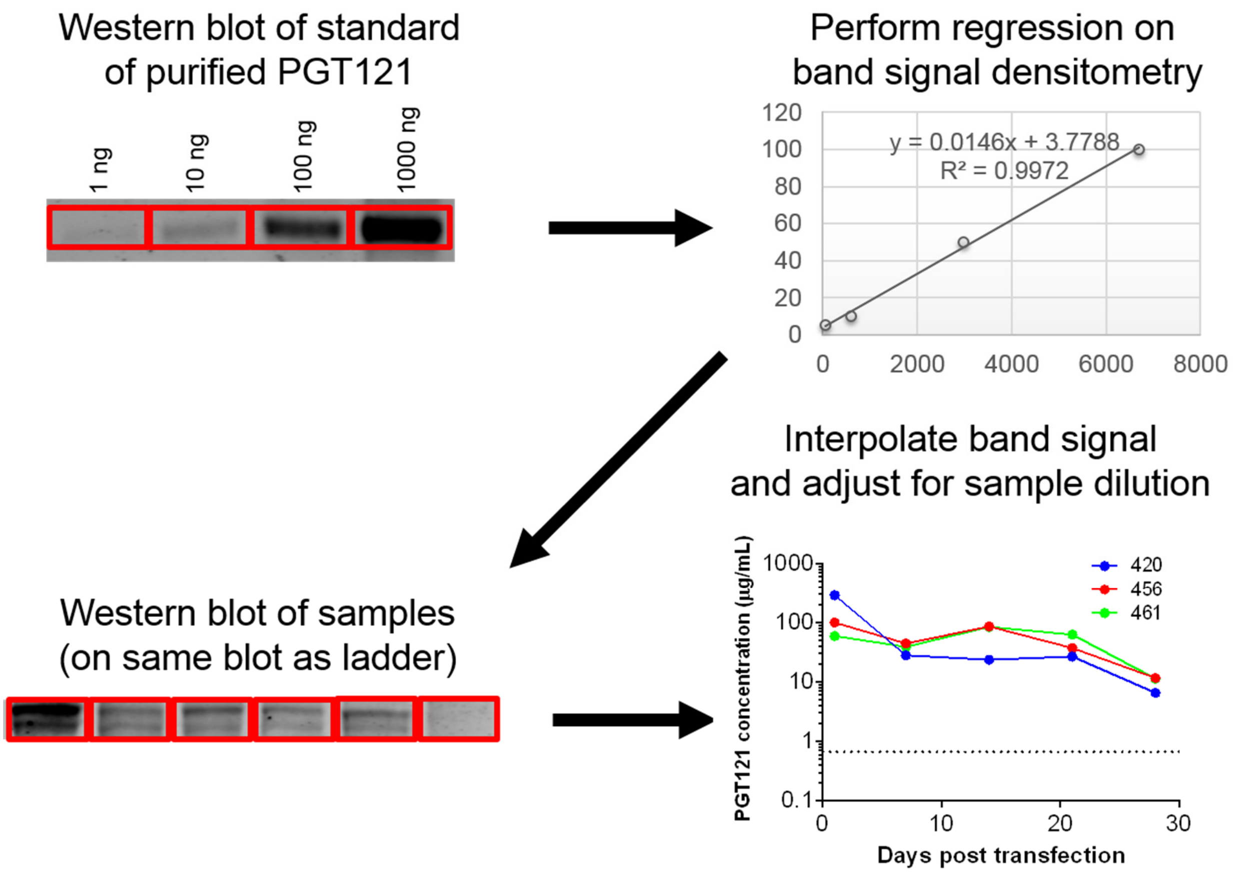


**Fig. S4. Quantitative Western methodology for aPGT121-NanoLuc.** Schematic of quantitative western blot protocol. On each gel, a standard of purified PGT121 was loaded and quantified by densitometry. A linear regression was then run on these values, and the densitometry of the samples was interpolated to determine the amount of PGT121 in the loaded sample. Samples were loaded onto the same gel in all cases.

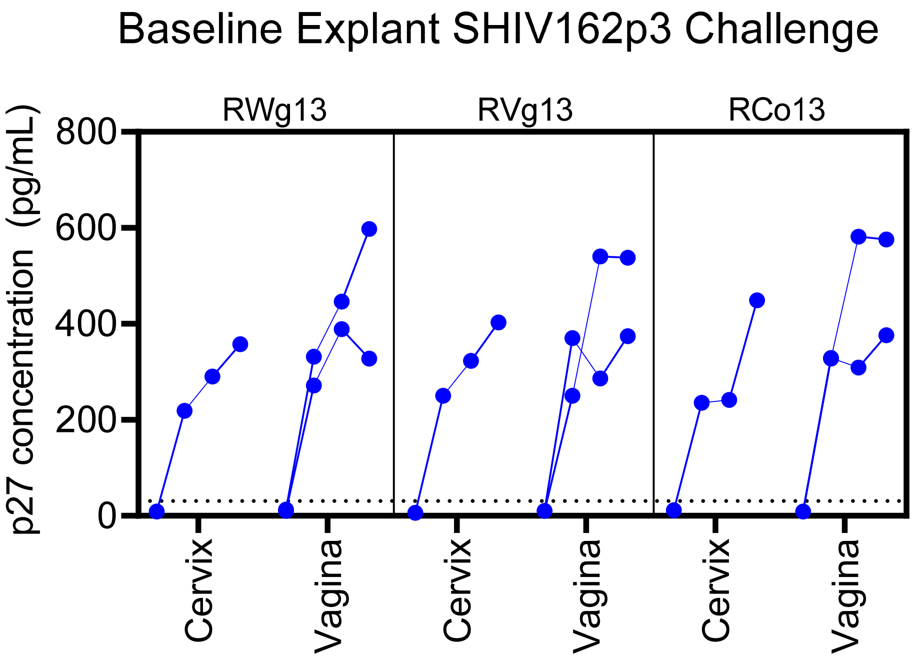


**Fig. S5. Baseline vaginal explant challenges.** Cervical and vaginal biopsies taken prior to mRNA delivery were challenged with SHIV162p3. Points indicate TZM assays on days 0, 4, 7, and 10 for each biopsy.

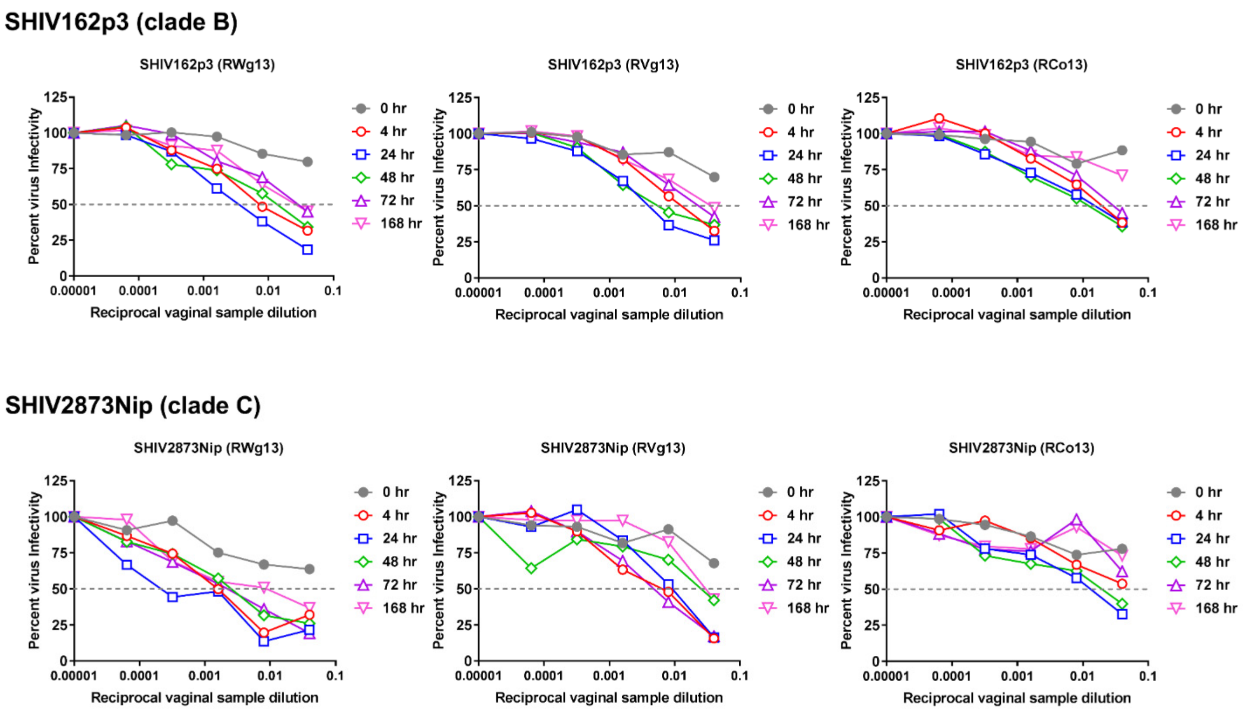


**Fig. S6. Vaginal secretion neutralization dilution series.** The neutralization activity of genital secretions from animals treated with 250 µg of aPGT121 mRNA at 4 h, 24 h, 48 h, 72 h, and 1-week post-transfection against Clade B and C SHIV strains was evaluated using the *in vitro* TZM-bl assay. Macaque RCo13 was only transfected with aPGT121 heavy chain (i.e. no light chain).

| | aPGT121 | | | | sPGT121 | | |
|---|---|---|---|---|---|---|---|
| | Animal ID | | | | Animal ID | | |
| Day | 420 | 456 | 461 | Mean | 401 | 414 | Mean |
| 1 | 594.0 | 122.0 | 299.0 | 338.3 | 22.1 | 100.7 | 61.4 |
| 7 | 60.5 | 51.6 | 80.1 | 64.1 | 26.1 | 46.1 | 36.1 |
| 14 | 91.5 | 68.1 | 97.7 | 85.7 | 13.8 | 12.8 | 13.3 |
| 21 | 52.2 | 70.8 | 149.0 | 90.6 | 11.1 | 8.4 | 9.8 |
| 28 | 35.6 | 55.2 | 41.1 | 44.0 | | | |

**Table S1: PGT121 concentrations by animal and mean in sheep secretions (µg/mL)**

| **Construct** | **Sequence** |
|---|---|
| **PGT121 LC-NanoLuc mRNA Geneblock** | CGATTGGCGGAAGGCCGTCAAGGCCGCATTTTTAAGCTTTAATACGAC<br>TCACTATAGGGAAATAAGAGAGAAAAGAAGAGTAAGAAGAAATATA<br>AGAGCCACCATGAAGTGGGTGACCTTCATCAGCCTGCTGTTCCTGTTC<br>AGCAGCGCCTACAGCAGCGACATCAGCGTGGCCCCCGGCGAGACCGC<br>CAGAATCAGCTGCGGCGAGAAGAGCCTGGGCAGCAGAGCCGTGCAGT<br>GGTACCAGCACAGAGCCGGCCAGGCCCCCAGCCTGATCATCTACAAC<br>AACCAGGACAGACCCAGCGGCATCCCCGAGAGATTCAGCGGCAGCCC<br>CGACAGCCCCTTCGGCACCACCGCCACCCTGACCATCACCAGCGTGGA<br>GGCCGGCGACGAGGCCGACTACTACTGCCACATCTGGGACAGCAGAG<br>TGCCCACCAAGTGGGTGTTCGGCGGCGGCACCACCCTCACCGTGCTGA<br>GAGCCGTGGCCGCCCCCAGCGTGTTCATCTTCCCCCCCAGCGAGGACC<br>AGGTGAAGAGCGGCACCGTGAGCGTGGTGTGCCTGCTGAACAACTTC<br>TACCCCAGAGAGGCCAGCGTGAAGTGGAAGGTGGACGGCGTGCTGAA<br>GACCGGCAACAGCCAGGAGAGCGTGACCGAGCAGGACAGCAAGGAC<br>AACACCTACAGCCTGAGCAGCACCCTGACCCTGAGCAGCACCGACTA<br>CCAGAGCCACAACGTGTACGCCTGCGAGGTGACCCACCAGGGCCTGA<br>GCAGCCCCGTGACCAAGAGCTTCAACAGAGGCGAGTGCGGAGGCGGG<br>GGCAGCGTCTTCACACTCGAAGATTTCGTTGGGGACTGGCGACAGAC<br>AGCCGGCTACAACCTGGACCAAGTCCTTGAACAGGGAGGTGTGTCCA<br>GTTTGTTTCAGAATCTCGGGGTGTCCGTAACTCCGATCCAAAGGATTG<br>TCCTGAGCGGTGAAAATGGGCTGAAGATCGACATCCATGTCATCATCC<br>CGTATGAAGGTCTGAGCGGCGACCAAATGGGCCAGATCGAAAAAATT<br>TTTAAGGTGGTGTACCCTGTGGATGATCATCACTTTAAGGTGATCCTG<br>CACTATGGCACACTGGTAATCGACGGGGTTACGCCGAACATGATCGA<br>CTATTTCGGACGGCCGTATGAAGGCATCGCCGTGTTCGACGGCAAAA<br>AGATCACTGTAACAGGGACCCTGTGGAACGGCAACAAAATTATCGAC<br>GAGCGCCTGATCAACCCCGACGGCTCCCTGCTGTTCCGAGTAACCATC<br>AACGGAGTGACCGGCTGGCGGCTGTGCGAACGCATTCTGGCGTGATA<br>AGCTGCCTTCTGCGGGGCTTGCCTTCTGGCCATGCCCTTCTTCTCTCCC<br>TTGCACCTGTACCTCTTGGTCTTTGAATAAAGCCTGAGTAGGAAGGCG<br>GCCGCAAAAACTGGGCC |

| | |
|---|---|
| **DAF GPI anchored PGT121 HC mRNA DNA IVT Template** | TTTTAAGCTTTAATACGACTCACTATAGGGAAATAAGAGAGAAAAGA<br>AGAGTAAGAAGAAATATAAGAGCCACCATGAAATGGGTCACCTTTAT<br>CAGCCTGCTGTTCCTGTTCAGCAGCGCCTACAGCCAGATGCAGCTCCA<br>AGAGTCTGGCCCTGGCCTGGTCAAGCCTAGCGAAACACTGAGCCTGA<br>CCTGTTCCGTGTCCGGCGCCAGCATCAGCGATAGCTACTGGTCCTGGA<br>TCAGAAGAAGCCCCGGCAAAGGCCTGGAATGGATCGGCTACGTGCAC<br>AAGAGCGGCGACACCAACTATAGCCCCAGCCTGAAGTCCAGAGTGAA<br>CCTGAGCCTGGACACCAGCAAGAACCAGGTGTCCCTGTCTCTGGTGGC<br>CGCCACAGCTGCTGATAGCGGCAAGTACTACTGCGCCAGAACACTGC<br>ACGGCAGACGGATCTATGGCATCGTGGCCTTCAACGAGTGGTTCACCT<br>ACTTCTACATGGACGTGTGGGGCAACGGCACCCAAGTGACAGTGTCT<br>AGCGCCAGCACAAAGGGCCCTAGCGTTTTCCCACTGGCTCCCAGCAGC<br>AGAAGCACCAGCGAATCTACAGCCGCTCTGGGCTGCCTCGTGAAGGA<br>CTACTTTCCTGAGCCAGTGACCGTGTCCTGGAACAGCGGCTCTCTGAC<br>ATCTGGCGTGCACACCTTTCCAGCCGTGCTGCAAAGCAGCGGCCTGTA<br>CTCTCTGAGCAGCGTGGTCACAGTGCCTAGCTCTAGCCTGGGCACCCA<br>GACCTACGTGTGCAATGTGAATCACAAGCCCAGCAACACCAAGGTGG<br>ACAAGAGAGTGGAAATCAAGACCTGCGGCGGAGGCAGCAAGCCTCCT<br>ACATGTCCTCCATGTCCTGCTCCAGAAGCTGCCGGCGGACCTTCCGTG<br>TTTCTGTTCCCTCCAAAGCCTAAGGACACCCTGATGATCAGCAGAACC<br>CCTGAAGTGACCTGCGTGGTGGTGGACGTGTCCCAAGAGGACCCCGA<br>CGTGAAGTTCAATTGGTACGTGAACGGCGCCGAGGTGCACCACGCTC<br>AGACAAAGCCAAGAGAGACACAGTACAACAGCACCTACAGAGTGGT<br>GTCCGTGCTGACCGTGACACACCAGGATTGGCTGAACGGCAAAGAGT<br>ACACCTGTAAAGTCTCCAACAAGGCCCTGCCTGCTCCTATCCAGAAAA<br>CCATCAGCAAGGACAAGGGCCAGCCTCGCGAACCCCAGGTTTACACA<br>CTGCCTCCAAGCAGAGAGGAACTGACCAAAAATCAGGTTTCCCTGAC<br>CTGCCTGGTTAAGGGCTTCTACCCCAGCGACATCGTGGTGGAATGGGA<br>GTCTAGCGGACAGCCCGAGAACACCTACAAGACCACACCTCCAGTGC<br>TGGACAGCGACGGCAGCTACTTCCTGTACAGCAAGCTGACAGTGGAC<br>AAGTCCAGATGGCAGCAGGGCAACGTGTTCAGCTGCAGCGTGATGCA<br>CGAGGCCCTGCACAACCACTACACCCAGAAGTCTCTGAGCGTCAGCC<br>CTGGCAAGCACGAGACAACCCCTAACAAAGGCAGCGGCACCACCTCT<br>GGCACCACAAGACTGCTGTCTGGCCACACCTGTTTCACACTGACCGGC<br>CTGCTGGGCACACTGGTTACAATGGGACTGCTGACCTGATAAGCTGCC<br>TTCTGCGGGGCTTGCCTTCTGGCCATGCCCTTCTTCTCTCCCTTGCACC<br>TGTACCTCTTGGTCTTTGAATAAAGCCTGAGTAGGAAGGCGGCCGCAA<br>AAA |

**Table S2: DNA template sequences used for *in vitro* transcription of mRNA**

| Primer Name | Purpose | Sequence |
|---|---|---|
| **A1: FWD** | **PCR Gibson Overlaps onto PGT121 LC - NanoLuc ORF** | GCGACGATTGGCGGAAGGCCGTCAAGGCCGCATTTTTAAG |
| **A2: RVS** | | CGGGCAGTGAGCGGAAGGCCCATGAGGCCCAGTTTTTG |
| **B1: FWD** | **Create linear pMA-7 vector template for subsequent Gibson cloning** | GGCCTTCCGCTCACTGCC |
| **B2: RVS** | | GGCCTTCCGCCAATCGTC |
| **C1: FWD** | **Sanger sequencing of insert fidelity of pMA-7 cloning** | TAAAACGACGGCCAGTGAGCGCGAC |
| **C2: RVS** | | CTGGCACGACAGGTTTCCCGACTGG |

**Table S3: Primers used during cloning of *in vitro* transcription templates**

**Movie S1: 3D PET/CT reconstruction of radiolabeled mRNA 24hrs post vaginal aerosol delivery.** Macaque RVg13 received 250 µg of $^{64}$Cu labelled PGT121 mRNA in two doses, once at the cervix, and another dose 3-4 cm caudally, at the vagina. Contrast levels were set to allow visualization of the relatively weak signal within the draining lymph nodes. Green represents low signal and purple represents high signal. 3D Reconstruction was created using Amira (Thermo Fisher).
